# Novel GBS-Based SNP Markers for Finger Millet and Their Use in Genetic Diversity Analyses

**DOI:** 10.3389/fgene.2022.848627

**Published:** 2022-04-26

**Authors:** Haftom Brhane, Teklehaimanot Haileselassie, Kassahun Tesfaye, Rodomiro Ortiz, Cecilia Hammenhag, Kibrom B. Abreha, Mulatu Geleta

**Affiliations:** ^1^ Institute of Biotechnology, Addis Ababa University, Addis Ababa, Ethiopia; ^2^ Department of Plant Breeding, Swedish University of Agricultural Sciences, Lomma, Sweden; ^3^ Ethiopian Biotechnology Institute, Ministry of Science and Technology, Addis Ababa, Ethiopia

**Keywords:** finger millet, gene diversity, genotyping-by-sequencing, single nucleotide polymorphism, tetraploid

## Abstract

*Eleusine coracana* (L.) Gaertn., commonly known as finger millet, is a multipurpose crop used for food and feed. Genomic tools are required for the characterization of crop gene pools and their genomics-led breeding. High-throughput sequencing-based characterization of finger millet germplasm representing diverse agro-ecologies was considered an effective method for determining its genetic diversity, thereby suggesting potential candidates for breeding. In this study, the genotyping-by-sequencing (GBS) method was used to simultaneously identify novel single nucleotide polymorphism (SNP) markers and genotype 288 finger millet accessions collected from Ethiopia and Zimbabwe. The accessions were characterized at individual and group levels using 5,226 bi-allelic SNPs, with a minimum allele frequency (MAF) of above 0.05, distributed across 2,500 scaffolds of the finger millet reference genome. The polymorphism information content (PIC) of the SNPs was 0.23 on average, and a quarter of them have PIC values over 0.32, making them highly informative. The grouping of the 288 accessions into seven populations based on geographic proximity and the potential for germplasm exchange revealed a narrow range of observed heterozygosity (Ho; 0.09–0.11) and expected heterozygosity (He) that ranged over twofold, from 0.11 to 0.26. Alleles unique to the different groups were also identified, which merit further investigation for their potential association with desirable traits. The analysis of molecular variance (AMOVA) revealed a highly significant genetic differentiation among groups of accessions classified based on the geographic region, country of origin, days to flowering, panicle type, and Al tolerance (*p* < 0.01). The high genetic differentiation between Ethiopian and Zimbabwean accessions was evident in the AMOVA, cluster, principal coordinate, and population structure analyses. The level of genetic diversity of finger millet accessions varies moderately among locations within Ethiopia, with accessions from the northern region having the lowest level. In the neighbor-joining cluster analysis, most of the improved cultivars included in this study were closely clustered, probably because they were developed using genetically less diverse germplasm and/or selected for similar traits, such as grain yield. The recombination of alleles *via* crossbreeding genetically distinct accessions from different regions of the two countries can potentially lead to the development of superior cultivars.

## Introduction

*Eleusine coracana* (L.) Gaertn. (finger millet) is an annual crop that belongs to the Chloridoideae subfamily of the Poaceae grass family. There are nine species under the genus *Eleusine* Gaertn., among which *E. coracana* is the only domesticated species (Bisht and Mukai, 2001, 2002). According to archeological records, finger millet was first cultivated in Ethiopia and Uganda (Hilu and De Wet, 1976). Thereafter, the crop was distributed to other African and Asian countries (Hilu and De Wet, 1976; Fakrudin et al., 2004; Goron and Raizada, 2015). Finger millet is an important cereal crop cultivated for its nutritious grains. In Africa, it is mainly grown in Ethiopia, Zimbabwe, Tanzania, Malawi, Zaire, Zambia, and Kenya (National Research Council, 1996). In Ethiopia, it is commonly grown across the whole Tigray, in parts of Amhara (Gonder and Gojjam), Oromia (Wellega, IIluababora, and Hararghe), and Southern Nations and Nationalities and People’s region (Gamo-Gofa) (Admassu et al., 2009). More than 2000 diverse finger millet accessions collected from its major cultivation areas in Ethiopia and abroad are conserved *ex situ* at the Ethiopian Biodiversity Institute (EBI; https://www.ebi.gov.et/) and used for research and breeding purposes. In Zimbabwe, it is a major crop in regions IV and V, which are the driest agro-ecological regions in the country (Mukarumbwa and Mushunje, 2010). The climatic conditions of these regions are similar to those of areas in Ethiopia where finger millet is widely cultivated.

Finger millet is a disomic tetraploid (*2n = 4x = 36*; AABB genome) crop. The diploid species *E. indica* (L.) Gaertn. (*2n = 2x = 18*; AA genome) and *E. floccifolia (Forssk.) Spreng* (*2n = 2x = 18*; BB genome) are considered the genome donors of *E. coracana* (Bisht and Mukai, 2001). It exhibits a great deal of phenotypic diversity for different traits. These include diversity in grain color (dark brown, light brown, radish brown, and white), growth habit (erect, recumbent, and prostrate), panicle shape (open, semi-curved, and curved), and flowering time (Upadhyaya et al., 2007). About 33 million hectares are estimated to be devoted to the production of different millet crops globally, with an estimated production quantity of 32.7 million tons of millet grains (FAOSTAT, 2020). About 4.5 million hectares of finger millet is produced globally every year (Antony Ceasar et al., 2018), and hence finger millet accounts for about 13.6% of the production of all millets. Ethiopia’s total finger millet harvested area is 480,511 ha (FAOSTAT, 2020), which produced an estimated 1.2 million tons of grains (FAOSTAT, 2020). Finger millet is consumed as porridge, pancake, injera, and traditional alcoholic beverages in Ethiopia (Bezaweletaw et al., 2006). It is favored because it is rich in nutrients (e.g., calcium, iron, thiamine, riboflavin, and nicotinic acid) and health-promoting components (tackle bone-related diseases, anemia, and cholesterol) and has better performance under stressful conditions (such as drought, disease, pest, salt, and soil acidity) (Barbeau and Hilu, 1993; Chethan and Malleshi, 2007; Sri et al., 2007; Nakarani et al., 2021a). It is a multipurpose crop because the grains are used for food, and its straw serves as a preferred animal feed (Chethan and Malleshi, 2007; Nakarani et al., 2021a).

Even though finger millet is an important food security, health-promoting, and climate-resilient crop, its productivity in Ethiopia is low due to various factors. The attention given to finger millet research and breeding has been insufficient and modern technologies were not well adopted to increase the efficiency of its production. Due to the highly limited availability of improved cultivars or their potential limitations, farmers are widely using local landraces. Consequently, major factors such as threshing difficulty, weeds, lodging, soil acidity, and blast disease caused by *Magnaporthe grisea* are limiting finger millet production and productivity, especially in Ethiopia (Erenso et al., 2007; Molla, 2010; Kebede et al., 2019). Aluminum is phytotoxic to plants through disrupting their root system, increasing the rigidity of cell wall and cell membrane, interrupting cell division, inducing oxidative stress, and blocking the influx of essential nutrients (Delhaize and Ryan, 1995; Zhang et al., 2014; Eekhout et al., 2017). Hence developing widely adaptable stable cultivars tolerant to the aforementioned biotic and abiotic factors to promote its production and productivity.

Plant breeding relies on the selection of diverse germplasm with desirable characteristics to develop new cultivars, achieved through the understanding of the genetic differences between germplasm used for breeding. Hence, the development and use of genome-wide markers are highly desirable to reveal the genetic diversity within a crop gene pool, followed by efficient use in conservation and plant breeding. Quantifying the genetic diversity of finger millet using such genome-wide DNA markers provides valuable information to breeders who assist in developing cultivars with various desirable traits, such as high yield and stress tolerance. Genotyping-by-sequencing (GBS) is a widely used next-generation sequencing (NGS) method developed for the simultaneous discovery of new markers and genotyping of target germplasm (Elshire et al., 2011). It is a high-throughput and cost-effective method used in various crops for different applications (Elshire et al., 2011; Poland and Rife, 2012; Huang et al., 2014; Alipour et al., 2017; Geleta et al., 2020; Hammenhag et al., 2020). The most popular DNA markers generated through the GBS method are single nucleotide polymorphism (SNP) markers. SNP markers are the most abundant sequence variation across crop genomes, which are suitable for analysis of genetic variation, population structure, marker-trait association, genomic selection, mapping quantitative trait loci (QTL), map-based cloning, and other plant breeding applications that need genome-wide scanning (Batley and Edwards, 2007; Kumar et al., 2012; Tsehay et al., 2020). The genetic diversity analysis of finger millet using GBS-derived SNP markers has received insufficient attention, despite its potential for providing markers useful for genetic improvement of the crop. The present study aimed at genotyping genetically diverse finger millet germplasm sampled from diverse agro-ecologies in Ethiopia and Zimbabwe using the GBS method as a means to develop new genomic resources for finger millet and molecular characterization of the germplasm.

## Materials and Methods

### Plant Material

This study used two hundred eighty-eight finger millet accessions composed of 274 landrace accessions (228 from Ethiopia and 46 from Zimbabwe) and 14 improved cultivars released in Ethiopia. The 228 Ethiopian landrace accessions were originally collected from Amhara (130), Benishangul-Gumuz (2), Oromia (51), Southern Nations, Nationalities and Peoples’ Region (SNNPR) (4), Tigray (32), and unknown locations in Ethiopia (9) (Supplementary Table S1). Landraces were obtained from the Ethiopian Biodiversity Institute (EBI), whereas the improved cultivars were obtained from Bako Agricultural Research Center (BARC).

### Testing Accessions for Aluminum Stress Tolerance

Aluminum (Al^3+^) stress tolerance of the accessions was initially screened using the hydroponic nutrient solution. The solution containing 500 µM KNO_3_, 500 µM CaCl_2_, 500 µM NH_4_NO_3_, 150 µM MgSO_4_.7H_2_O, 10 µM KH_2_PO_4_, 2 µM FeCl_3_ (III), and 75 µM Al_2_ (SO_4_)_3_.18H_2_O was prepared according to Zhou et al. (2013). The pH of the nutrient solution was adjusted to 4.3 (using 1 M HCl or NaOH) and renewed every day to maintain the pH and Al^3+^ concentration relatively constant. For this experiment, similar-sized seeds from each accession were selected and surface-sterilized by soaking in 3% sodium hypochlorite solution for 5 min and rinsed thoroughly with water. The sterilized seeds of each accession were then wrapped in tissue paper, moistened with distilled water, and placed in a Petri dish for 36 h under dark conditions to initiate germination. Then, the seeds of each accession were grouped into two, and one group was grown in the hydroponic nutrient solution containing 100 μM Al. In contrast, the other group was grown as a control in the nutrient solution without aluminum for 10 days. This was followed by measuring the root length of the seedlings in cm using a ruler and calculating the relative root length (RRL) in percent as follows:

$$\mathrm{RRL}\;\left(\%\right)=\frac{\mathrm{Root}\;\mathrm{length}\;\mathrm{under}\;\mathrm{Al}\;\mathrm{treatment}}{\mathrm{Root}\;\mathrm{length}\;\mathrm{under}\;\mathrm{control}}\times 100\%.$$

### Planting, Leaf Tissue Sampling, and Phenotyping

In order to ensure at least one germinating seeds of each of the 288 accessions, five seeds were planted in a 5 L plastic pot for each accession in a greenhouse at the Swedish University of Agricultural Sciences (SLU, Alnarp, Sweden). After seed germination, extra seedlings were removed, and only a single seedling was maintained for each accession. Three weeks after planting, the leaf tissue of each accession was separately sampled using the BioArk Leaf collection kit from LGC, Biosearch Technologies (https://biosearchassets.blob.core.windows.net/assetsv6/guide_bioark-leaf-collection-kit.pdf), as described in Osterman et al. (2021). Afterward, the samples were sent to LGC, Biosearch Technologies (Berlin, Germany) for genomic DNA extraction, followed by GBS-based genotyping. The plants were hand-weeded and treated with pesticides and fertilizers during their growth in the greenhouse. Afterward, data on days to flowering and panicle shape were recorded.

### DNA Extraction, Genotyping-By-Sequencing Optimization, Sequencing, and Read Pre-Processing

A high molecular weight genomic DNA of the 288 accessions was extracted using the Sbeadex plant kit (https://biosearch-cdn.azureedge.net/assetsv6/sbeadex-plant-data-sheet.pdf) for the GBS analysis. For the construction of a GBS library, *Pst*l (CTGCA*G, a six-base cutter) and *Ape*Kl (G*CWGC, a four-base cutter) restriction enzymes were used following the recommendations of the experts at LGC, Biosearch Technologies, who optimized restriction enzymes for various crops. The combined use of *Pst*l-*Ape*Kl produced a fragment size distribution suitable for sequencing on Illumina platforms with a mean insert size of ∼220 bp. The GBS was conducted in 2 × 150 bp (paired-end) sequencing mode using NexSeq 500/550 v2 and NovaSeq SP FC NGS platforms to generate the reads. This produced about 1.5 million read pairs per sample. The reads were then adapter clipped, and those containing Ns were discarded along with reads whose 5′ ends did not match the restriction enzyme site. This was followed by trimming the 3′-end of the remaining reads using Trimmomatic v. 0.3 (Bolger et al., 2014) to obtain an average Phred quality score of ≥20 over a window of ten bases and discarding reads with a final length <20 bp.

### Read Alignment, Single Nucleotide Polymorphism Discovery and Filtering, and Genotype Calling

For this GBS analysis, *Eleusine coracana* subsp. *coracana* cultivar ML-365 scaffold-level genome assembly (Hittalmani et al., 2017; https://www.ncbi.nlm.nih.gov/assembly/GCA_002180455.1/) was used as a reference genome, as there is no chromosome-level assembly available for finger millet yet. BWA-MEM v. 0.7.12 software package (Li and Durbin, 2009) was used to align quality trimmed reads against the reference genome. The mapping rate of the GBS reads to the reference genome (containing 525,627 scaffolds with a total size of 1.3 Gb) was 99.16%. Then, Freebayes v. 1.2.0 (Garrison and Marth, 2012) was used for variant discovery and genotype calling as diploids. The alignment of the reads from the 288 finger millet genotypes resulted in the discovery of 101,889 SNPs. The SNPs were then filtered using GBS-specific criteria (minimum read count > 8, minimum allele frequency (MAF) ≥ 0.05, and percentage of samples with assigned genotype ≥66%). Out of 71,140 SNPs with average read counts of above eight, 11,120 SNPs were fully covered (2 × 150 bp reads), had MAF of ≥5%, and scored in ≥66% of the samples. The 11,120 SNPs spanned 4,729 scaffolds, with 51.4% of the scaffolds containing a single SNP, although up to 32 SNPs were recorded per scaffold (Supplementary Figure S1). The 11,120 SNPs were further filtered, and 5,226 biallelic SNPs with genotypic data across all samples (no missing data) were used for genetic diversity analyses described below.

### Data Analysis

The vast majority of the finger millet accessions used in this study are landraces, originally collected from diverse agro-ecologies. Additionally, each accession was represented by a single genotype as described above. Hence, to facilitate data analysis and better understand the pattern of genetic diversity, the accessions were grouped based on different criteria prior to data analyses. First, the 288 accessions were grouped into seven groups according to their geographic origin, which will be referred to as “populations” (Pop-1 to Pop-7) from here on for the sake of simplicity. Pop-1 to Pop-5 represent Ethiopian landrace accessions, and their descriptions are as follows: Pop-1 represents accessions collected from Agew-Awi, Gojam, Bahrdar, and Metekel (northwestern accessions); Pop-2 represents accession from western Tigray and Gonder (northern accessions); Pop-3 represents accessions collected from Wellega and IIluababora (western accessions); Pop-4 represents accessions from central, eastern, and southern Tigray and northern Wello (northeastern accessions); Pop-5 represents accessions with unknown sampling location; and Pop-6 represents the 14 improved cultivars whereas the 46 Zimbabwean landrace accessions form Pop-7.

Second, the accessions were grouped based on days to flowering, as early flowering and late flowering types (Supplementary Figure S2). Third, the accessions were grouped based on their panicle shape as open (fingers straight), semi-compact (top of fingers curved inward), and compact (fingers fully curved inward) (Supplementary Figure S3). Fourth, the accessions were broadly grouped into two groups based on the performance of accessions against aluminum toxicity (Al), where accessions having relative root length (RRL) above and below 50% were grouped as Al-tolerant and Al-susceptible, respectively (Supplementary Figure S4). Finger millet production is hampered by abiotic factors such as Al toxicity, so it is necessary to use this information to group the accessions according to their level of Al tolerance.

The genetic diversity of the 288 accessions grouped based on different criteria was assessed using different statistical programs. For this data analysis, 5,226 SNPs with minor allele frequency (MAF) above 5% and no missing values were used. Polymorphism information content (PIC) of the SNPs was calculated as described by Hildebrand et al. (1994). The frequency spectrum of segregating sites of each population was analyzed using the command analysis through segregating sites option in population size changes added to a toolbar of DNASP v 6 (Rozas et al., 2003). The number of alleles (Na), effective number of alleles (Ne), observed heterozygosity (Ho), expected heterozygosity (He), unbiased expected heterozygosity (uHe), Shannon’s information index (I), percentage of polymorphic loci (PPL), and number of private alleles were calculated using GeneAlEx v. 6.51b2 (Peakall and Smouse, 2006). The analysis of molecular variance (AMOVA) and Hardy–Weinberg equilibrium test were performed using Arlequine v. 3.5.2.2 (Excoffier and Lischer, 2010). The average number of pairwise differences within and among the populations and Nei’s genetic distance (Nei, 1972) were also calculated using the same software, and their graphic representation was generated using scripts of a console version of the R statistical package (Rcmd) triggered through a command button added to Arlequin’s toolbar.

Neighbor-joining (NJ) cluster analysis was done based on Nei’s genetic distance using the MEGA7 program (Kumar S. et al., 2016). GeneAlEx v. 6.51b2 was used for principal coordinate analysis (PCoA). Furthermore, the genetic structure of the accessions was evaluated using STRUCTURE 2.3.4 software (Pritchard et al., 2000). STRUCTURE analysis was run for K = 1 to K = 10 with 10 independent runs at each K using the admixture model with burn-in period length of 1,000,000 and a Markov chain Monte Carlo of 1,000,000 replications. STRUCTURESELECTOR (Li and Liu, 2018) was used to determine the optimum K based on the different approaches described by Puechmaille (2016). After the optimum K was determined, the CLUMPACK beta version (Kopelman et al., 2015) was used to display the graphical representation of the population structure.

## Results

### Characteristics and Distribution of the Single Nucleotide Polymorphism Markers

**FIGURE 1:**
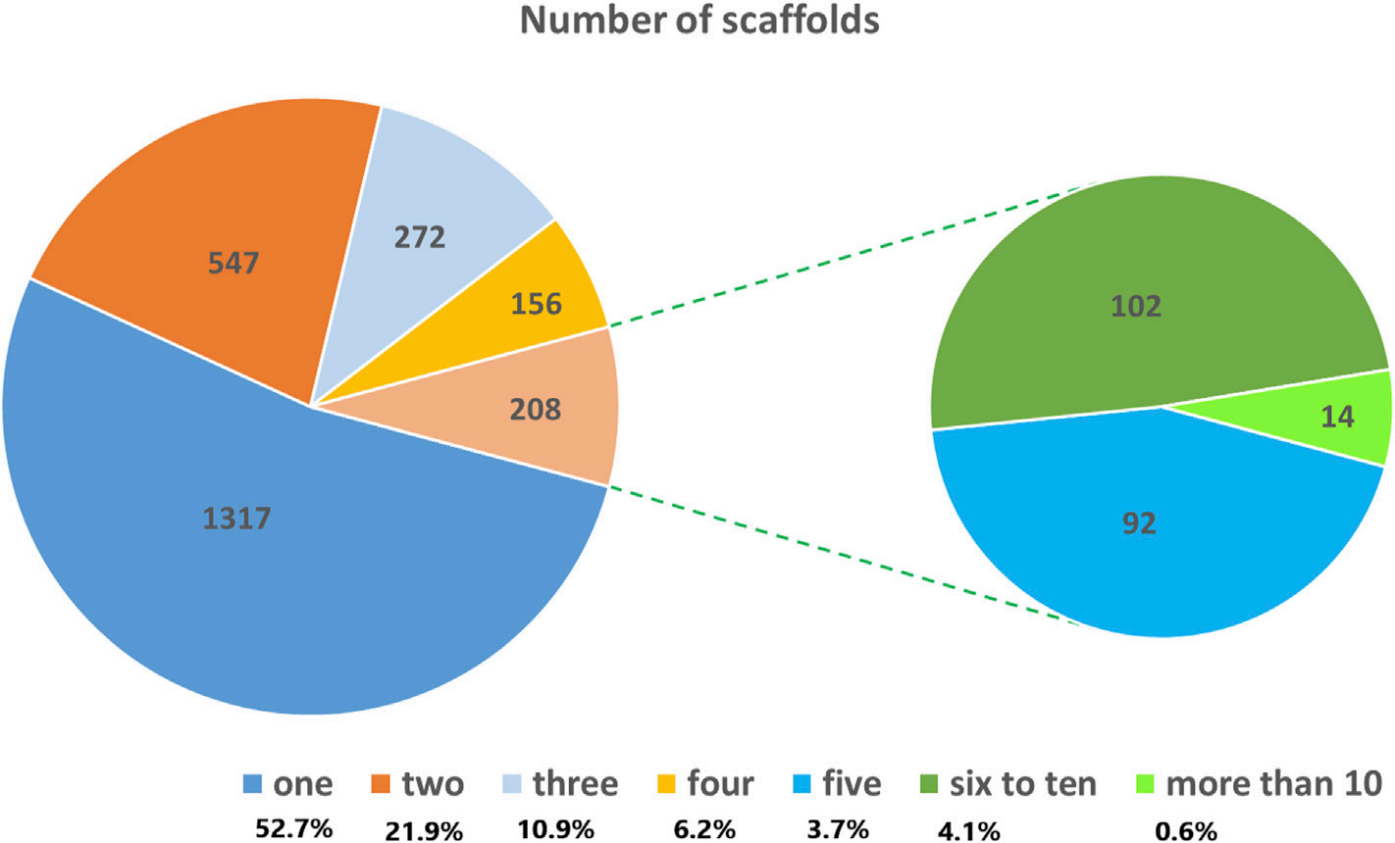
Pie chart showing the distribution of the 5,226 SNPs used for genetic diversity analysis across the scaffolds of the finger millet reference genome.

**FIGURE 2:**
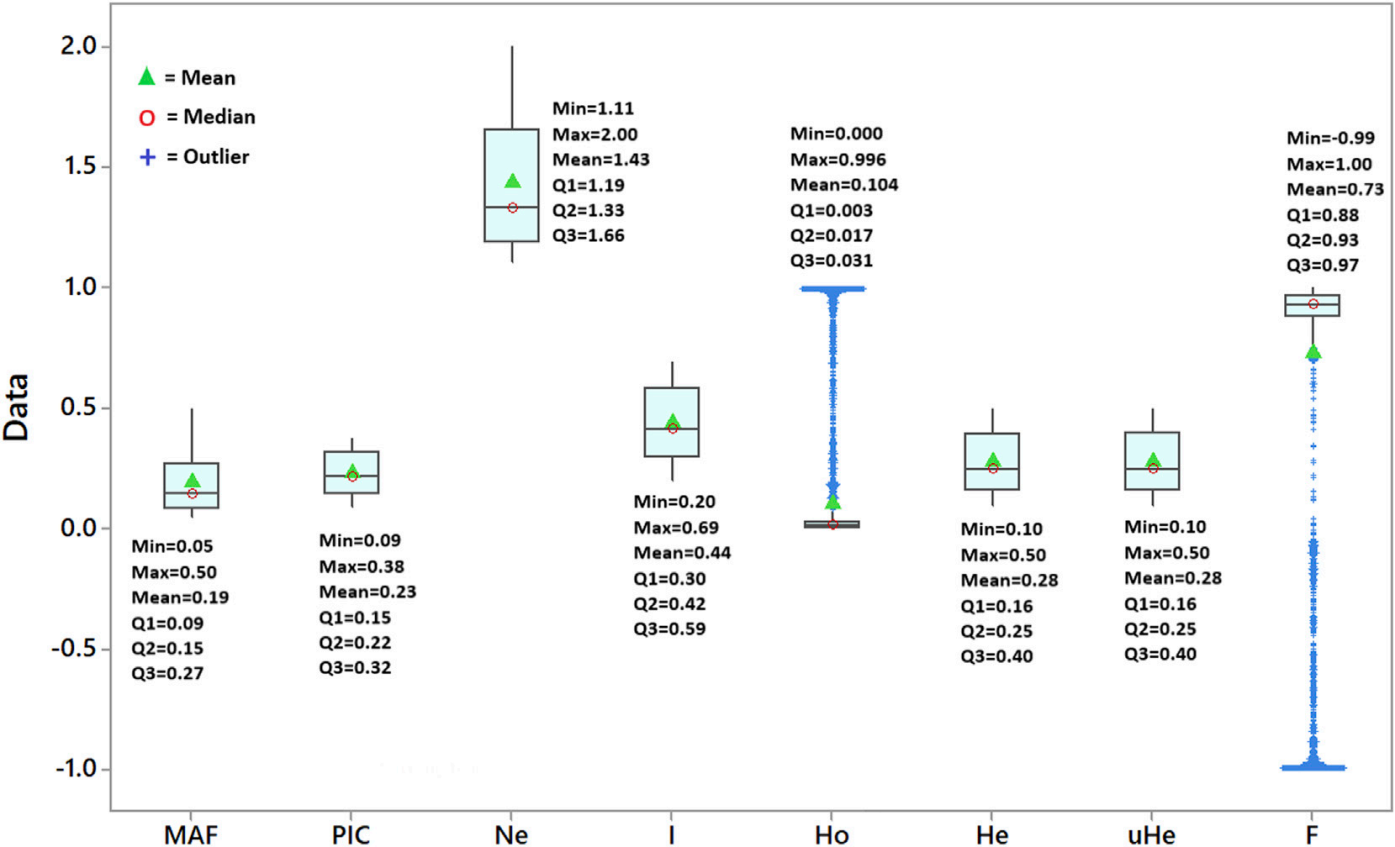
Box plot depicting the variation in minimum allele frequency (MAF), polymorphism information content (PIC), effective number of alleles (Ne), Shannon information index (I), observed heterozygosity (Ho), expected heterozygosity (He), unbiased expected heterozygosity (uHe), and fixation index (F) of the 5,226 SNP loci across the 288 accessions.

**FIGURE 3:**
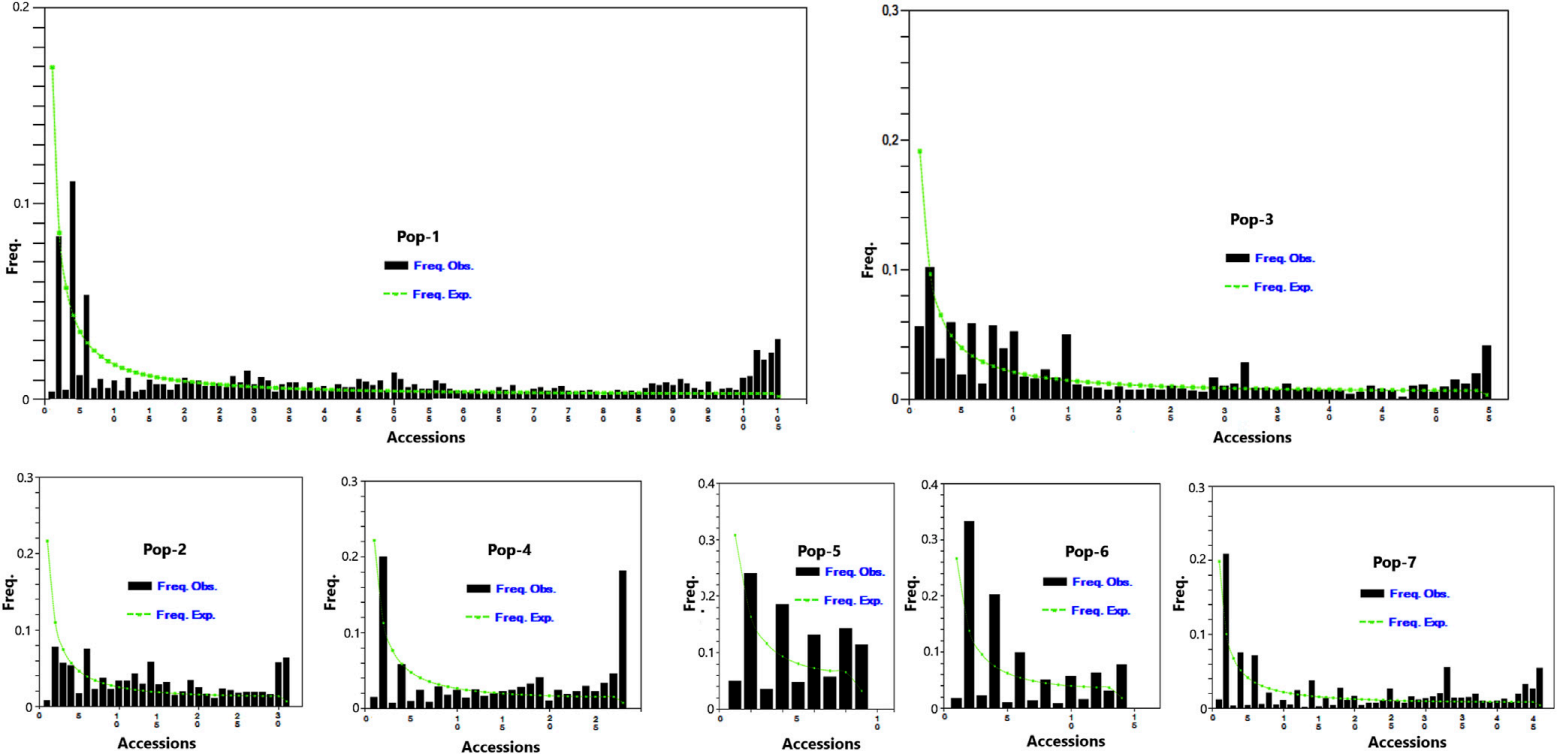
Graph depicting minor allele frequency (MAF) based site-frequency spectrum across 5,226 SNP loci in the seven finger millet populations.

A total of 5,226 SNP markers with MAF above 5% and without missing data were used for genetic diversity analyses of the finger millet accessions (Supplementary Table S2). The 5,226 SNPs were spread across 2,500 scaffolds, with 52.7% of these scaffolds containing only one of the SNPs although the number of SNPs per scaffold ranged from 1 to 18 (Figure 1). The mean and median of the MAF of the SNPs were 0.19 and 0.15, respectively, with 25% of the SNPs having a MAF above 0.27 (Figure 2). The polymorphism information content (PIC) of the SNPs varied from 0.09 to 0.38 with a mean and third quartile of 0.23 and 0.32, respectively. Similarly, the mean values of the effective number of alleles (Ne), Shannon information index (I), observed heterozygosity (Ho), expected heterozygosity (He), unbiased expected heterozygosity (uHe), and fixation index (F) of the SNPs were 1.43, 0.44, 0.10, 0.28, 0.28, and 0.73, respectively (Figure 2). A significant number of SNPs were outliers in terms of Ho and F. The Ho of 75% of the SNPs was below 0.03, whereas the F of the SNPs varied from −0.99 to 1.0 (Figure 2). Based on the site-frequency spectrum, it was revealed that the MAF distribution of the SNP loci varied substantially among the seven finger millet populations (Figure 3). Only 4.5% of the individuals across all populations had minor alleles at more than 10% of the SNP loci, with all individuals having minor alleles at less than 35% of the loci. At a population level, only 1.0%, 1.8%, 0%, 7.1%, 55.6%, 21.4%, and 2.2% of the individuals had minor alleles in ≥10% of the SNP loci in Pop-1 to Pop-7, respectively. A coalescent analysis based on the expected site-frequency spectrum did not match the observed frequency distributions well, with a vast majority of individuals in all populations exhibiting lower expected values than observed (Figure 3).

**FIGURE 4:**
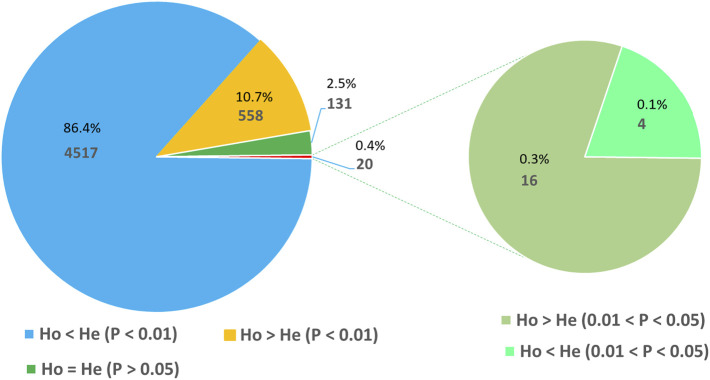
Pie chart illustrating the number and proportion of SNP loci at Hardy–Weinberg equilibrium (HWE) and those with a significant deviation from HWE in terms of heterozygote excess and deficiency.

The Hardy–Weinberg equilibrium (HWE) test was conducted using 1000,000 steps in the Markov chain and 100,000 steps in dememorization by assuming that the 288 accessions are random samples from a single population. This analysis revealed that 2.5% of the loci were at HWE, 11% were with excess heterozygosity, and 86.5% were with heterozygosity deficit. Interestingly, the vast majority of the loci deviated from HWE at a highly significant level (*p* < 0.01) with 10.7% and 86.4% of them showing heterozygosity excess and deficit, respectively (Figure 4).

### Genetic Diversity of the Finger Millet Accessions

As a means to facilitate data analysis and determine patterns of genetic diversity, the 288 accessions were divided into seven populations based on geographic proximity and the potential for germplasm exchange. Pop-1 to Pop-7 comprised 105, 31, 55, 28, 9, 14, and 46 accessions, respectively. Based on their flowering time under greenhouse conditions, we broadly grouped the accessions into “early” and “late” flowering types, with 115 days to flowering as the dividing point (Supplementary Figure S2). The early and late flowering types accounted for 69.4 and 30.6% of the accessions, respectively. In terms of panicle shape (Supplementary Figure S3), the majority of the accessions had open panicles (71.2%), whereas those with curved and semi-curved panicles accounted for 19.1% and 9.7% of the accessions, respectively. The Al tolerance experiment revealed that 20.8% of the accessions had RRL values above 50% and were hence considered tolerant of Al toxicity. However, the RRL values of the vast majority of accessions (77.2%) were below 50% and so were considered susceptible to Al toxicity. The results of genetic diversity analyses for the different groups of accessions are presented below.

Among the seven populations, the effective number of alleles (Ne) varied from 1.19 (Pop-4) to 1.44 (Pop-1), with an overall mean of 1.33 (Table 1). Shannon information index (I) ranged from 0.16 (Pop-4) to 0.40 (Pop-1) with an average value of 0.30. In terms of observed heterozygosity (Ho), there were slight differences between the populations, which only varied from 0.09 to 0.11. On the contrary, expected heterozygosity (He) or gene diversity ranged from 0.11 (Pop-4) to 0.26 (Pop-1) with an average value of 0.20, which were similar to the corresponding values of unbiased expected heterozygosity (uHe). The percentage of polymorphic loci (PPL) showed threefold variation among the populations, with Pop-1 having the highest (96%) and Pop-4 the lowest (32%) values, and on average 69% of the loci were polymorphic. Interestingly, the level of genetic diversity of the improved cultivars (Pop-6) was similar to the overall average values regardless of the relatively small sample size (Table 1). Pop-1, Pop-2, Pop-3, and Pop-7 were more diverse and Pop-4 and Pop-5 less diverse when compared with Pop-6 (improved cultivars).

**TABLE 1:** Estimates of different genetic diversity parameters for different groups of finger millet accessions: mean values for the observed number of alleles (N_A_), effective number of alleles (Ne), Shannon’s information index (I), observed heterozygosity (Ho), expected heterozygosity (He), unbiased genetic diversity (uHe), percentage of polymorphic loci (PPL), and number of private alleles (NPA).

Groups	N	Na	Ne	I	Ho	He	uHe	PPL	NPA
Pop-1^a^	105	1.96	1.44	0.40	0.11	0.26	0.26	0.96	13
Pop-2^a^	31	1.69	1.36	0.33	0.10	0.22	0.22	0.69	0
Pop-3^a^	55	1.87	1.33	0.32	0.11	0.20	0.21	0.87	0
Pop-4^a^	28	1.32	1.19	0.16	0.09	0.11	0.11	0.32	0
Pop-5^a^	9	1.53	1.33	0.28	0.10	0.19	0.20	0.53	0
Pop-6^a^	14	1.69	1.31	0.31	0.09	0.19	0.20	0.69	0
Pop-7^a^	46	1.76	1.36	0.32	0.09	0.21	0.21	0.76	11
Mean	41.1	1.69	1.33	0.30	0.10	0.20	0.20	0.69	3.4
EF^b^	126	2.00	1.45	0.43	0.10	0.28	0.28	1.00	25
LF^b^	162	2.00	1.42	0.40	0.11	0.26	0.26	1.00	1
Mean	144	2.00	1.43	0.42	0.10	0.27	0.27	1.00	13
Compact^c^	55	1.70	1.38	0.33	0.09	0.22	0.22	0.70	64
Semi-compact^c^	31	1.87	1.34	0.33	0.11	0.21	0.21	0.87	0
Open^c^	202	1.99	1.40	0.38	0.11	0.25	0.25	0.99	111
Mean	96	1.85	1.37	0.35	0.10	0.23	0.23	0.85	58.3
Tolerant^d^	60	1.93	1.45	0.41	0.10	0.27	0.27	0.93	11
Susceptible^d^	228	2.00	1.39	0.39	0.11	0.24	0.24	1.00	387
Mean	144	1.96	1.42	0.40	0.10	0.26	0.26	0.96	199

EF, early flowering; LF, late flowering; N, number of accessions.

a Accessions were grouped according to geographic proximity and the potential for germplasm exchange.

b Accessions were grouped according to days to flowering.

c Accessions were grouped according to panicle shape.

d Accessions were grouped according to tolerance to aluminum toxicity.

The genetic analyses of the accessions grouped based on panicle type revealed that accession with open panicle had higher mean values for most of the diversity parameters (Na = 1.99, Ne = 1.40, I = 0.38, Ho = 0.11, He = 0.25, and PPL = 100%) than semi-compact accessions (Na = 1.87, Ne = 1.34, I = 0.33, Ho = 0.11, He = 0.21, and PPL = 87%) and compact accessions (Na = 1.70, Ne = 1.38, I = 0.33, Ho = 0.09, He = 0.22, and PPL = 70%) (Table 1). The early and late flowering groups had quite similar values for the different genetic diversity estimates, with the early flowering group having Ne = 1.45, I = 0.43, Ho = 0.10, He = 0.28, and PPL = 100% and the late flowering group having Ne = 1.42, I = 0.40, Ho = 0.11, He = 0.26, and PPL = 100% (Table 1). The Al-tolerant group had slightly higher values in effective number of alleles, Shannon information index, and expected heterozygosity (Ne = 1.45, I = 0.41, and He = 0.27) than the Al-susceptible group (Ne = 1.39, I = 0.39, and He = 0.24). All loci were polymorphic in the Al-susceptible group as opposed to 93% polymorphic loci in the Al-tolerant group. Among the seven populations, private alleles were recorded only in Pop-1 (NPA = 13) and Pop-7 (NPA = 11) (Table 1). The frequency of the unique alleles in Pop-1 varied from 0.138 to 0.148, while in Pop-7, it varied from 0.315 to 0.576 (Table 2). In the case of panicle-type group, the number of unique alleles to open, semi-compact, and compact panicle groups were 111, 0, and 64, respectively. Moreover, 25 unique alleles were specific to early flowering group as opposed to only one in late flowering group. Likewise, 11 and 387 private alleles were recorded in the Al-tolerant and Al-susceptible groups, respectively (Table 1).

**TABLE 2:** List of SNP loci with a private allele in Pop-1 and Pop-7 with their alleles and allele frequencies.

No.	Locus name	Population	Private allele	Private allele frequency
1	LXGH01033309.1_4802	Pop-1	T	0.148
2	LXGH01036535.1_25361	Pop-1	G	0.138
3	LXGH01036535.1_25481	Pop-1	G	0.138
4	LXGH01224870.1_62858	Pop-1	C	0.138
5	LXGH01296805.1_22457	Pop-1	T	0.138
6	LXGH01296805.1_22532	Pop-1	A	0.138
7	LXGH01296805.1_22641	Pop-1	G	0.138
8	LXGH01296805.1_22649	Pop-1	G	0.138
9	LXGH01296805.1_4809	Pop-1	A	0.138
10	LXGH01296805.1_4820	Pop-1	G	0.138
11	LXGH01405315.1_45826	Pop-1	G	0.138
12	LXGH01405315.1_45892	Pop-1	C	0.138
13	LXGH01405315.1_45929	Pop-1	G	0.138
14	LXGH01094451.1_9437	Pop-7	T	0.435
15	LXGH01151768.1_642	Pop-7	T	0.315
16	LXGH01174315.1_91057	Pop-7	A	0.576
17	LXGH01211987.1_24770	Pop-7	G	0.348
18	LXGH01246444.1_13014	Pop-7	A	0.391
19	LXGH01321131.1_13891	Pop-7	A	0.348
20	LXGH01321131.1_14094	Pop-7	C	0.348
21	LXGH01321131.1_14706	Pop-7	C	0.348
22	LXGH01321131.1_14787	Pop-7	C	0.359
23	LXGH01321131.1_16828	Pop-7	A	0.348
24	LXGH01482256.1_2202	Pop-7	T	0.380

### Genetic Variation Within and Among Different Groups of Accessions

Analysis of molecular variance (AMOVA) was conducted to estimate the genetic variation within and among different groups of finger millet accessions grouped based on different criteria. AMOVA for the seven populations revealed that the variation within and among the populations accounted for 70% and 30% of the total variation, respectively (Table 3). When accessions were grouped based on country of origin (Ethiopia vs. Zimbabwe), AMOVA revealed that the variation within and among countries accounted for 57.1% and 42.9% of the total variation, respectively. Similarly, the variation among days to flowering groups (early vs. late), panicle-type groups (open vs. semi-compact vs. compact), and aluminum tolerance groups (Al-tolerant vs. Al-susceptible) accounted for 30%, 9.3%, and 28% of the corresponding total variations, respectively (Table 3). Interestingly, the variations among the groups formed based on all criteria were highly significant (*p* < 0.01). The estimates for the differentiation among the groups (F_ST_) were 0.31 (for the seven populations), 0.43 (for country of origin), 0.30 (for panicle type), 0.09 (for days to flowering), and 0.28 (for aluminum tolerance) (Table 3).

**TABLE 3:** Analysis of molecular variance (AMOVA) of different groups formed from the 288 finger millet accessions based on their geographic origin, country of origin, panicle type, days to flowering, and aluminum tolerance using 5,226 SNP markers.

Source of variation	df	SS	MS	Est. Var.	PV	FI	*p*-value
Seven populations							
Among pops	6	183,071.9	30,511.98	767.44	30	0.305	*p* < 0.001
Within pops	281	492,412.4	1,752.357	1752.36	70		
Total	287	675,484.3		2519.80			
Ethiopia vs. Zimbabwe							
Among countries	1	115,499.5	115,499.5	1,468.74	42.9	0.429	*p* < 0.001
Within countries	286	559,984.8	1,957.989	1,957.99	57.1		
Total	287	675,484.3		3,426.73			
Open vs. semi-compact vs. compact panicles							
Among ear shape groups	2	62,139.14	31,069.57	453.09	30	0.300	*p* < 0.001
Within ear shape groups	285	301,174.6	1,056.753	1,056.75	70		
Total	287	363,313.7		1,509.85			
Early vs. Late flowering							
Among flowering groups	1	34,807.07	34,807.07	229.73	9.3	0.09	*p* < 0.001
Within flowering groups	286	641,277.1	2,242.228	2,242.23	90.7		
Total	287	676,084.2		2,471.96			
Al-tolerant vs. Al-susceptible							
Among tolerance groups	1	42,568.6	42,568.6	436.29	28	0.280	*p* < 0.001
Within tolerance groups	286	320,745.1	1,121.486	1,121.49	72		
Total	287	363,313.7		1,557.77			

DF, degrees of freedom; SS, sum of squares; MS, mean square; Est. Var, estimated variance; PV, percentage of variation; FI, fixation index (F_ST_).

### Pairwise Genetic Distance, Cluster, and Principal Coordinate Analyses

**FIGURE 5:**
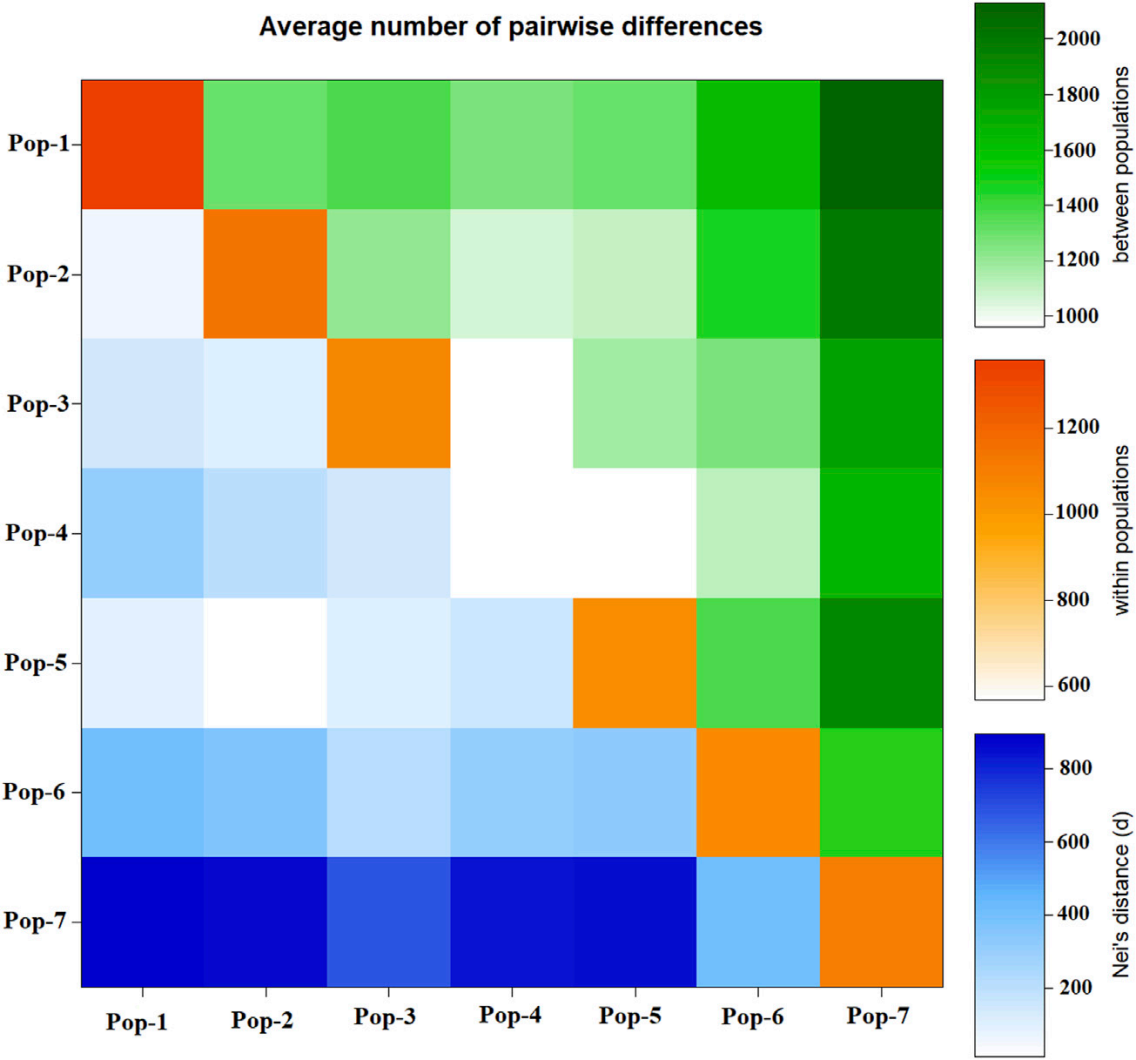
Graphical representation of the average number of pairwise differences between the seven finger millet populations generated using 5,226 SNP markers. Below diagonal represents Nei’s genetic distance, diagonal represents pairwise differences within populations, and above diagonal represents pairwise differences between populations.

Pairwise Nei’s standard genetic distance among the seven finger millet populations ranged from 0.01 to 0.48. The lowest genetic distance was found between Pop-2 and Pop-5 (0.01), followed by Pop-1 vs. Pop-2 (0.04) and Pop-1 vs. Pop-5 (0.06) (Table 4), which is in agreement with the average number of pairwise differences (Figure 5). The highest genetic distance was obtained between Pop-4 and Pop-7 (0.48), followed by Pop-2 vs. Pop-7 (0.44) (Table 4). Pop-7 (representing Zimbabwean accessions) had a higher genetic distance to most of the finger millet populations from Ethiopia (0.27–0.48) when compared with the genetic distance between pairs of the Ethiopian populations (0.01–0.30). Pop-7 was more similar to Pop-6 (released cultivars) among the six populations representing the Ethiopian finger millet gene pool with a genetic distance of 0.27 (Table 4; Figure 5). The mean Nei’s genetic distance of each population from the other populations varied from 0.16 (Pop-3) to 0.40 (Pop-7). The analysis of the average number of pairwise differences within populations revealed that Pop-1 and Pop-4 had the highest and lowest differences, respectively (Figure 5). In agreement with the genetic distance data, most Zimbabwean accessions were phenotypically distinct from most Ethiopian accessions by being early flowering type and having compact panicles and shorter and stronger stems.

**TABLE 4:** Pairwise Nei’s standard genetic distance between the seven populations of finger millet evaluated using 5,226 SNP markers. A diagonal value is the mean genetic distance of the corresponding populations from the other populations.

	Pop-1	Pop-2	Pop-3	Pop-4	Pop-5	Pop-6	Pop-7
Pop-1	**0.18**						
Pop-2	0.04	**0.17**					
Pop-3	0.10	0.09	**0.16**				
Pop-4	0.20	0.19	0.14	**0.25**			
Pop-5	0.06	0.01	0.09	0.19	**0.17**		
Pop-6	0.24	0.24	0.16	0.30	0.24	**0.24**	
Pop-7	0.41	0.44	0.38	0.48	0.43	0.27	**0.40**

**FIGURE 6:**
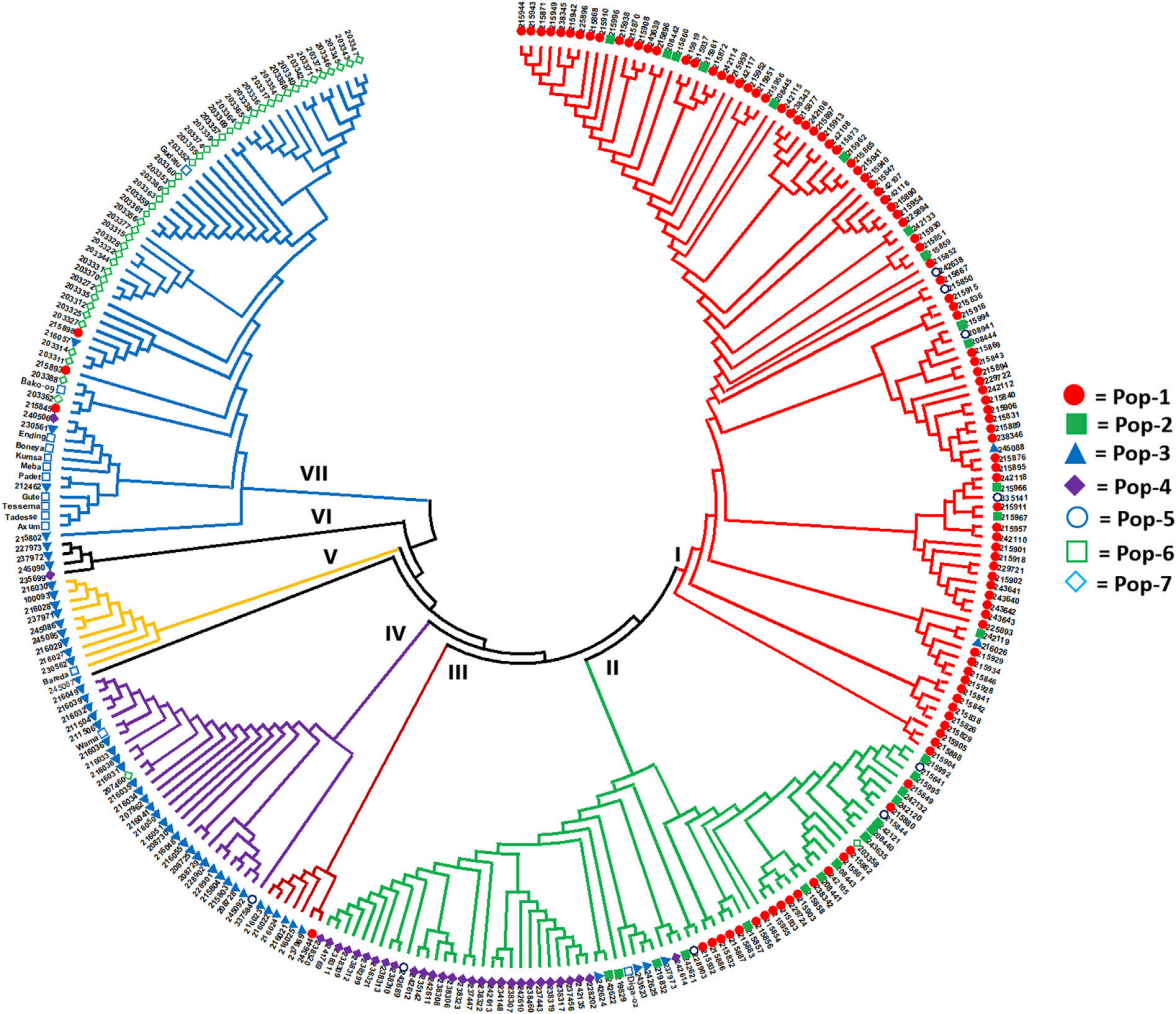
Nei’s standard genetic distance-based neighbor-joining tree showing the clustering pattern of the 288 finger millet accessions. Accessions sharing a symbol with the same shape and color belong to the same population.

The Nei’s standard genetic distance-based neighbor-joining (NJ) cluster analysis of the 288 accessions revealed seven clusters with a good clustering pattern according to their geographic origin, except that few accessions collected from different regions were clustered together (Figure 6). Cluster-I was the largest and more diverse group comprising 36 early-flowering and 67 late-flowering accessions from Pop-1 (84), Pop-2 (13), Pop-3 (two), and Pop-5 (four). Cluster-II was also a diverse group in terms of populations containing 38 early- and 33 late-flowering accessions from Pop-1 (17), Pop-2 (15), Pop-3 (four), Pop-4 (29), Pop-5 (four), Pop-6 (one), and Pop-7 (one). Cluster-III comprised one accession of Pop-1 and six accessions of Pop-3, all of which were a late flowering type. Cluster-IV is composed of 14 early-flowering and 15 late-flowering type, of which 26 accessions were from Pop-3, and the remaining three accessions were from Pop-5, Pop-6, and Pop-7. Cluster-V comprised 10 accessions (two early- and eight late-flowering type) from Pop-3 (nine) and Pop-6 (one). Cluster-VI was the smallest cluster with four late-flowering accessions from Pop-3 (three) and Pop-4 (one). Another large and heterogeneous group was Cluster-VII comprising 46 early- and 17 late-flowering accessions from Pop-1 (three), Pop-3 (four), Pop-6 (12), and Pop-7 (44) (Figure 6).

**FIGURE 7:**
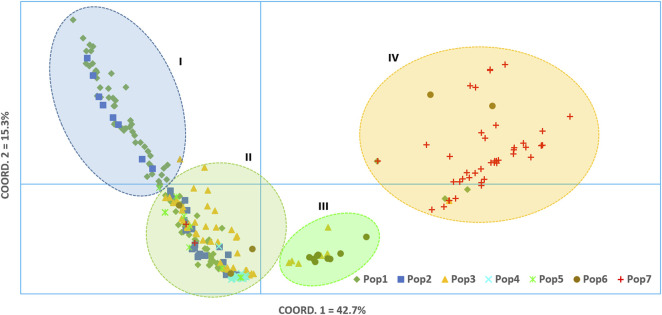
Bi-plot of principal coordinate analysis (PCoA) generated using 5,226 SNP markers depicting the genetic relationship among the 288 finger millet accessions. A symbol of the same shape and color represents accessions of the same population.

Principal coordinate analysis (PCoA) was used to visualize the differentiation among the 288 accessions (Figure 7). The analysis revealed that the first and second coordinates together explained 58% of the total variation. In spite of the lack of distinct clustering, the 288 accessions could be broadly categorized into four clusters, mainly based on the first coordinate that explained 42.7% of the total variation. Cluster-I comprised almost exclusively accessions from Pop-1 and Pop-2, only with additional two accessions from Pop-3 and Pop-5. Cluster-II was the most heterogenous group comprising accessions from all the seven populations. Interestingly, all accessions of Pop-4 were tightly grouped in this cluster. Cluster-III was the smallest, comprising six accessions of Pop-3 and nine accessions of Pop-6. The vast majority of accessions from Zimbabwe (Pop-7) were placed in Cluster-IV, containing three accessions from Pop-1, one accession from Pop-3, and two accessions from Pop-6 (Figure 7).

### Population Structure Analysis

**FIGURE 8:**
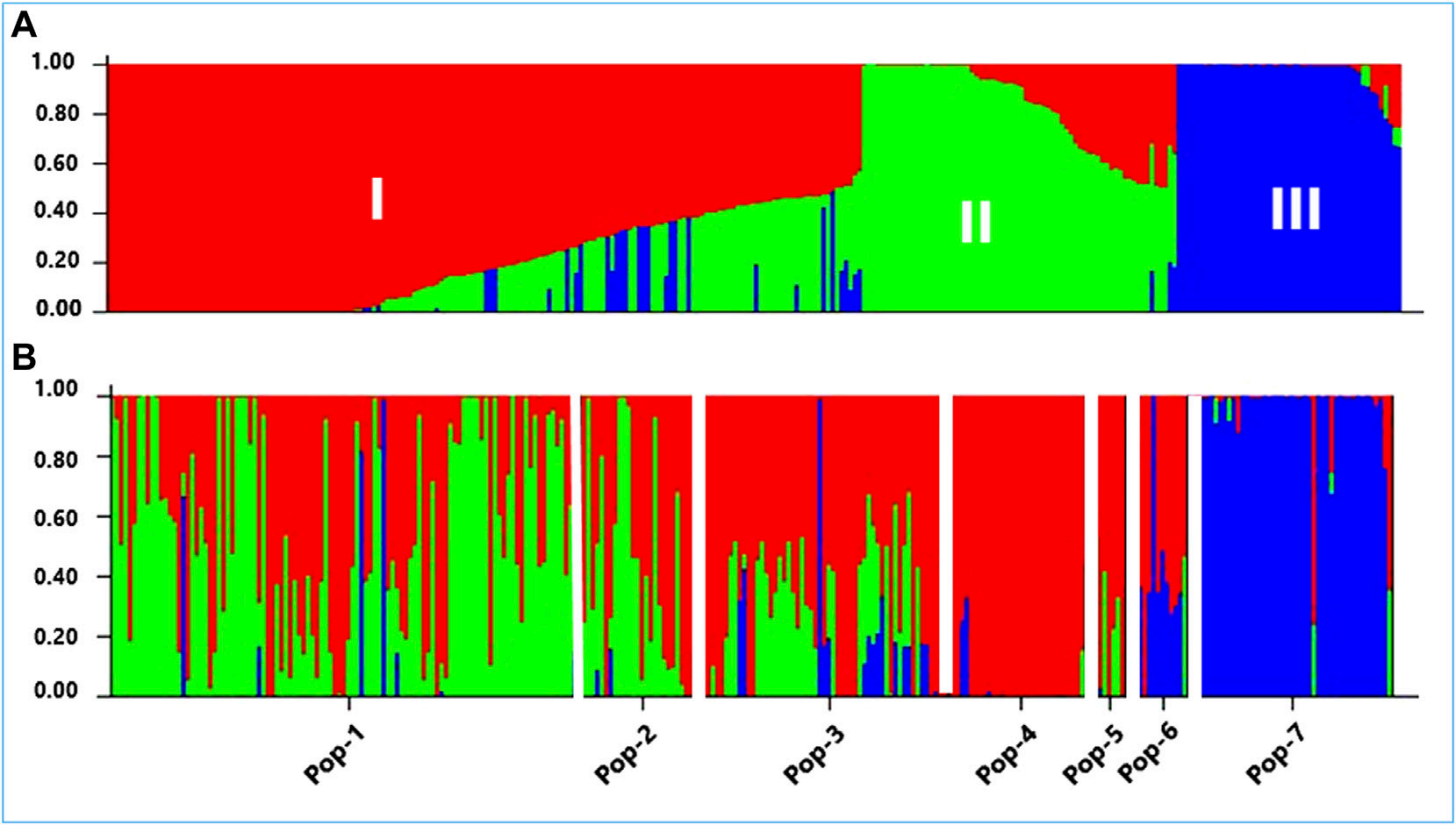
A graphical display of the genetic structure of 288 accessions of finger millet at K = 3, forming three clusters (shown by different colors) and exhibiting different levels of admixture. **(A)** Individual accessions were arranged according to the level of their membership in different clusters and **(B)** individual accessions were arranged according to their assigned populations (Pop-1 to Pop-7) comprising 105, 31, 55, 28, 9, 14, and 46 accessions, respectively.

According to the admixture model-based population genetic structure analysis conducted using the 5,226 SNP markers, three genetic populations represent the 288 accessions the best (Figure 8). This analysis revealed that Pop-1, Pop-2, Pop-3, and Pop-5 were dominated by alleles from the first two genetic populations (red and green). Population-4 was the least diverse among the Ethiopian finger millet populations, with its alleles almost entirely originating from the first genetic population (red). The improved cultivars appeared to be the results of strong admixture between the second and third genetic populations (green and blue). In agreement with the results of cluster analysis and PCoA, accessions from Zimbabwe were, in general, distinctly separated from the Ethiopian accessions by having the vast majority of their alleles from the third genetic population (blue) (Figure 8).

## Discussion

### The Single Nucleotide Polymorphism Markers

The GBS method that uses two restriction enzymes is a highly effective method for reducing genome complexity and generating high-quality SNP markers in several crops, including barley, wheat (Poland et al., 2012), and cacao (Osorio-Guarin et al., 2020), which was also demonstrated in the present study. The GBS method has been used previously for simultaneous SNP discovery and genotyping in finger millet (Kumar A. et al., 2016; Gimode et al., 2016; Qi et al., 2018; Tiwari et al., 2020). However, none of them used the *Pst*l/*ApeK*l methylation-sensitive restriction enzyme combination. Hence, the SNPs identified in this study represent a novel genomic resource for finger millet as the target genomic regions were different from those in the previous study. The final set of 5,226 SNP markers used in this study were distributed across 2,500 scaffolds with only one SNP per scaffold in most cases, indicating a wide distribution of these SNPs across the finger millet genome. Hence, the *Pst*l/*ApeK*l enzyme combination is suitable for GBS-based SNP discovery in finger millet and closely related species. The allele frequencies of most of these SNPs exceed 10%, with a quarter exceeding 27%, which makes them ideal for population genetics and genome-wide association studies (GWAS). The polymorphism information content (PIC) is a measure of the informativeness of molecular markers, and the higher the PIC value of a locus is, the more informative it is (Hildebrand et al., 1994; Shete et al., 2000). A bi-allelic SNP locus can have a maximum PIC value of 0.38, which is attained when both alleles are equally frequent. The SNP loci in the present study have an average PIC of 0.23, and a quarter have PIC values over 0.32, making them highly informative. The average PIC (0.23) obtained in this study was lower than the average PIC (0.29) value estimated for finger millet accessions in Gimode et al. (2016). The slightly higher mean PIC value in Gimode et al. (2016) could most likely be due to the use of more diverse accessions (wild and cultivated) and much fewer SNP loci (80) with more frequent alleles when compared to the present study (MAF > 0.05). Similarly, it is also lower than the average PIC of earlier studies using expressed sequence tags and genomic microsatellites (SSR) markers (Gimode et al., 2016; Krishna et al., 2018; Lule et al., 2018; Pandian et al., 2018; Brhane et al., 2021). The PIC values of SNP markers are generally lower than those of SSR markers, as the average number of alleles per locus is higher in SSRs than in bi-allelic SNPs (Chao et al., 2009).

Finger millet is a highly self-pollinating crop. Hence, the overall expected heterozygosity (He) is expected to be higher than the observed heterozygosity (Ho) if all other HWE assumptions are met. In line with the expectation, the mean He value (0.28) was higher than the mean Ho value (0.10) in the present study. A recent study in sorghum (a self-pollinating cereal crop) using 3001 SNP markers reported mean He and Ho values of 0.10 and 0.06 (Enyew et al., 2021). The lower values in the latter case are most likely because most of the SNP markers used were located within genes (Enyew et al., 2021) and hence are expected to be less diverse than GBS-based SNP markers. Similar bi-allelic SNP-based studies in noug (*Guizotia abyssinica*; Tsehay et al., 2020) and red clover (*Trifolium pratense*; Osterman et al., 2021) reported Ho values of 0.24 and 0.22, respectively. It is expected that noug and red clover have higher Ho values than finger millet and sorghum because both of these crops are strictly outcrossing, enabling a higher level of heterozygosity. According to the HWE test, 97.5% of the loci showed significant deviation from HWE, with 86.5% of loci showing heterozygote deficiency. Due to the self-pollinating nature of finger millet, there is a high likelihood of heterozygote deficiency at the vast majority of loci, although other evolutionary forces may have played a role as well. In contrast, approximately one-tenth of the SNP loci displayed excess heterozygosity, which is interesting. The result of the present study suggests that selection pressures that favor heterozygosity are at work at these loci, and further studies targeting these loci will shed more light on their significance. As indicated by Tsehay et al. (2020), loci with heterozygote excess or deficit contrary to the reproductive mechanism of a plant indicate that such loci are under various types and intensity of selection and other evolutionary forces.

### Genetic Diversity Within Groups of Accessions

The genetic characterization of germplasm using codominant DNA markers is a crucial step toward developing stable and productive cultivars with various desirable traits. In the present study, 5,226 GBS-based SNP markers were used to examine the pattern and level of genetic diversity across 288 finger millet accessions representing the finger millet gene pool in Ethiopia and Zimbabwe. According to the result of site-frequency spectrum analysis, there is substantial genetic diversity among the seven finger millet populations, as evidenced by variation in minor allele frequency distributions between them. However, individual accessions in different populations generally bear minor alleles at fewer loci compared to results obtained in a similar study in sorghum (Enyew et al., 2021). More than 50% of individuals in Pop-5 and over 20% of individuals in Pop-6 had minor alleles in at least 10% of the SNP loci, as opposed to the other populations with minor alleles in less than 8% of the loci. Pop-6 represents released cultivars, and hence it is likely that minor alleles play a significant role in their improved traits. Although Pop-5 lacks passport data, it would be interesting to investigate them further to uncover their phenotypic diversity of desirable traits.

The level of genetic diversity observed across the populations in this study was comparable to that of earlier studies in finger millet based on SSR markers (Babu et al., 2014; Nirgude et al., 2014; Ramakrishnan et al., 2016; Pandian et al., 2018). Among the seven finger millet populations, Pop-1 was the most diverse as estimated based on different parameters (Ne = 1.44; I = 0.40; He = 0.26; PPL = 0.96). Because the sample size of this population is larger than that of the other populations and the accessions originated from broader areas and diverse agro-ecological environments, their genetic diversity is expected to be higher. However, sample size does not fully explain the lowest genetic diversity recorded in Pop-4 as Pop-5 and Pop-6 had a smaller sample size than Pop-4. For example, the sample size of Pop-2 (northern accessions) and Pop-4 (northeastern accessions) is quite similar, but the gene diversity of the former was twice that of the latter, clearly indicating low genetic diversity in finger millet cultivated in the northeastern part of Ethiopia. In addition to its relatively high genetic diversity, Pop-1 (northwestern accessions) came on top in terms of the private alleles it harbors (13). Hence, the region represented by Pop-1 may be considered a hotspot for finger millet genetic diversity in Ethiopia, which is appropriate for the crop’s *in situ* conservation because finger millet is cultivated in large quantities in this region. The average values of different estimates of genetic variation of the four Ethiopian landrace populations (Pop-1 to Pop-4) were similar to those of the Zimbabwean landrace population (Pop-7). Hence, the finger millet gene pools in the two countries can be considered equally diverse. The level of genetic diversity of the population representing the cultivars (Pop-6) was within the diversity range of the landrace populations, which suggests that at least some cultivars were developed based on different finger millet genetic resources. It is noteworthy that the observed heterozygosity (Ho) of the cultivars was quite similar to that of the landraces, indicating that the cultivars are not pure lines. This indicates that their further improvement is possible through self-pollination.

Among the three groups of accessions classified based on the panicle types, the genetic diversity of those with open panicles was slightly higher than that of the other two groups. This could be mainly because of the sample size effects as accessions with open panicles accounted for two-thirds of the 288 accessions, representing wider geographic areas. Finger millet with open panicles is preferred for cultivation in Ethiopia due to its better grain yield, drought tolerance, resistance to bird attack and shattering, and ease to thresh (Tsehaye and Kebebew, 2002; Bezaweletaw et al., 2007). Hence, their higher genetic diversity is desirable for further improvement through breeding. On the contrary, slightly higher genetic diversity was recorded in the early flowering group than in the late flowering group, regardless of the smaller sample size used. Similarly, a slightly greater genetic diversity was found in the Al-tolerant group than in the Al-susceptible group, despite a roughly fourfold larger sample size in the latter. The accessions in the late-maturing group and Al-susceptible group were exclusively Ethiopian, whereas both Ethiopia and Zimbabwe were widely represented in the early maturing group and Al-tolerant group, which may explain in part the higher diversity level in the latter groups. Private alleles were found in both the early maturing and the Al-tolerant groups, and their potential associations with desirable characteristics require further investigation. Private alleles that are strongly associated with a desirable trait can be used in marker-assisted selection (MAS) and selection of parental lines for crossbreeding based development of superior cultivars with multiple desirable traits (Park et al., 2008). Early flowering and tolerance to aluminum toxicity are desirable traits in finger millet. Hence, based on landraces that combine early flowering and Al tolerance, improved cultivars with characteristics of interest can be developed, taking advantage of their genetic diversity.

### Genetic Relationships Among Accessions and Their Groups and Population Genetic Structure

Analysis of molecular variance (AMOVA) revealed highly significant genetic differentiations among groups of accessions classified based on the geographic region of origin, country of origin, flowering time, panicle shape, and Al tolerance (*p* < 0.01). The significant differentiation among groups of finger millet accessions was in agreement with the previously published research using EST and genomic SSR markers (Babu et al., 2014; Pandian et al., 2018; Brhane et al., 2021). In the present study, the highest level of differentiation was observed between Ethiopian and Zimbabwean accessions accounting for 43% of the total genetic variation (Table 2). The generally distinct differentiation between the two groups was also evident from the genetic distance data, average pairwise differences, cluster analysis, PCoA, and population structure (Table 3; Figures 5–8). Phenotypically, Zimbabwean accessions are dominated by early flowering plants with shorter and stronger stems, compact panicles, and better Al tolerance compared to the Ethiopian accessions. Accessions with desirable traits such as early flowering and Al tolerance exist in both groups. Hence, crossbreeding between selected finger millet genotypes from the two countries increases the likelihood of recombination of alleles that will eventually lead to superior cultivars. The Ethiopian accession 208440 and Zimbabwean accession 203347 are excellent examples in this regard as both are early flowering and Al-tolerant (Supplementary Table S1). However, the high genetic distance between them placed them separately in Cluster-II and Cluster-VII, respectively (Figure 6).

Eleven of the 14 released cultivars were clustered together in Cluster-VII, with nine of them showing tight clustering. A recent study based on SSR markers reported similar close clustering of finger millet cultivars (Brhane et al., 2021). The clustering of cultivars close together indicates that they were developed based on genetically similar germplasm and/or selected for similar traits during breeding. Because most Ethiopian cultivars clustered with the Zimbabwean landrace accessions, germplasm genetically similar to the latter may have been used in the development of the cultivars. Among the Ethiopian landrace accessions, those in Pop-3 (western accessions) appeared to be genetically closer to the cultivars, except for cultivar Diga-2 (Figure 6). Thus, finger millet cultivar development might have heavily relied on germplasm from western Ethiopia. Nevertheless, further research into the pedigrees of the cultivars and potential gene flow after their release are required to shed more light on this. The clustering pattern of accessions along the lines of days to flowering, aluminum tolerance, and panicle types did not resolve well in the cluster analysis and PCoA. However, accessions carrying the desirable characteristics of these traits are genetically diverse and can be improved through breeding due to potential transgressive segregations (Ortiz et al., 2020).

The cluster analysis and PCoA clearly showed that Pop-1 (northwestern accessions) and Pop-2 (northern accessions) were poorly differentiated, suggesting stronger gene flow between them. However, some of the accessions in these populations are different from those in other populations, as shown in Cluster-I of the PCoA (Figure 7). Pop-4 is quite unique in that most of its accessions are genetically highly similar (low genetic diversity) and show close genetic relationships with some members of all other populations, indicating its low genetic differentiation. The fact that accessions representing all populations were found within Cluster-II of PCoA strongly suggests a countrywide gene flow, albeit to a different degree. Overall, the clustering pattern of accessions generally agrees with a recent study on Ethiopian finger millet accessions based on genomic and EST-derived SSR markers (Brhane et al., 2021).

An important feature of the Bayesian statistical approach to population structure analysis is that it enables the identification of genotypes that originate purely from one genetic population or are the results of genetic admixture (Pritchard et al., 2000; Kumar A. et al., 2016). This study revealed that the 288 genotypes from diverse sources originate from three genetic populations (K = 3). Similar to the present study, population structure analyses in previous research on finger millet using SSR and SNP markers revealed three genetic groups with different levels of admixture (Dida et al., 2008; Kumar A. et al., 2016; Ramakrishnan et al., 2016; Lule et al., 2018; Pandian et al., 2018; Brhane et al., 2021). In the present study, the Zimbabwean accessions and Ethiopian accessions appeared to have originated from different genetic populations with few exceptions, which is expected due to the clear geographic separation between the two countries. In addition, it is interesting to note that the Zimbabwean accessions were much less admixed than the Ethiopian landrace accessions, except for those in Pop-4 (Figure 8). The genetic structure of Pop-1 and Pop-2 is highly similar, which indicates that gene flow is stronger between the geographic areas the accessions represent.

## Conclusion

In the present study, over ten thousand SNP markers were detected using GBS, providing a new genomic resource for finger millet. The characterization of 5,226 of these SNPs in diverse finger millet populations from Ethiopia and Zimbabwe shows that they are highly informative and, therefore, suitable for different applications, such as genome-wide association studies and population genetics. The observed heterozygosity among landraces and cultivars is low, agreeing with the crop’s reproductive mechanism. However, there was excess heterozygosity at about one-tenth of the SNP loci, although the crop is mainly self-pollinating, suggesting evolutionary forces favoring heterozygosity may be at play at these loci. Future research focusing on these loci will provide more insight into their role. In order to determine whether private alleles found in different accession groups have potential associations with desired traits, further investigation is needed. Within Ethiopia, finger millet landrace accessions from different geographic regions differ moderately in terms of genetic diversity, with those from northeastern Ethiopia being the least diverse. The fact that some accessions from different regions clustered closely together suggests a countrywide gene flow, though to a different extent. The genetic differentiation among accessions classified by geographic region, country of origin, days to flowering, panicle type, and Al tolerance is significantly high, with differentiation among countries being the highest. In the case of improved cultivars, most of them clustered tightly together, suggesting that they were developed from similar germplasm and/or selected for the same traits, mainly grain yield. In addition, the level of their heterozygosity suggests that they can be further improved through self-pollination-based breeding. By using genetically distinct accessions from different geographic regions or countries, crossbreeding can potentially lead to the development of superior cultivars due to the recombination of alleles.

## Data Availability

The datasets presented in this study can be found in online repositories. The names of the repository/repositories and accession number(s) can be found in the article/Supplementary Material the GBS reads: https://www.ncbi.nlm.nih.gov/bioproject/?term=PRJNA791522.

## References

[B1] Admassu S. Teamir M. Alemu D. (2009). Chemical Composition of Local and Improved finger Millet [*Eleusine Corocana* (L.) Gaetrtin] Varieties Grown in Ethiopia. Ethiopian J. Health Sci. 19.

[B2] Alipour H. Bihamta M. R. Mohammadi V. Peyghambari S. A. Bai G. Zhang G. (2017). Genotyping-by-sequencing (GBS) Revealed Molecular Genetic Diversity of Iranian Wheat Landraces and Cultivars. Front. Plant Sci. 8, 1293. 10.3389/fpls.2017.01293 28912785 PMC5583605

[B3] Antony Ceasar S. Maharajan T. Ajeesh Krishna T. P. Ramakrishnan M. Victor Roch G. Satish L. (2018). Finger Millet [*Eleusine Coracana* (L.) Gaertn.] Improvement: Current Status and Future Interventions of Whole Genome Sequence. Front. Plant Sci. 9, 1054. 10.3389/fpls.2018.01054 30083176 PMC6064933

[B4] Arora A. Kundu S. Dilbaghi N. Sharma I. Tiwari R. (2014). Population Structure and Genetic Diversity Among Indian Wheat Varieties Using Microsatellite (SSR) Markers. Aust. J. Crop Sci. 8.

[B5] Babu B. K. Agrawal P. K. Pandey D. Kumar A. (2014). Comparative Genomics and Association Mapping Approaches for Opaque2 Modifier Genes in finger Millet Accessions Using Genic, Genomic and Candidate Gene-Based Simple Sequence Repeat Markers. Mol. Breed. 34, 1261–1279. 10.1007/s11032-014-0115-2

[B6] Barbeau W. E. Hilu K. W. (1993). Protein, Calcium, Iron, and Amino Acid Content of Selected Wild and Domesticated Cultivars of finger Millet. Plant Food Hum. Nutr. 43, 97–104. 10.1007/BF01087914 8475005

[B7] Batley J. Edwards D. (2007). “SNP Applications in Plants,” in Association Mapping in Plants (Springer), 95–102. 10.1007/978-0-387-36011-9_6

[B8] Bezaweletaw K. Sripichitt P. Wongyai W. Hongtrakul V. (2006). Genetic Variation, Heritability and Path-Analysis in Ethiopian finger Millet [*Eleusine Corocana* (L.) Gaetrtin] Landraces. Agric. Nat. Resour. 40, 322–334.

[B9] Bezaweletaw K. Sripichitt P. Wongyai W. Hongtrakul V. (2007). Phenotypic Diversity of Ethiopian finger Millet [*Eleusine Coracana* (L.) Gaertn] in Relation to Geographical Regions as an Aid to Germplasm Collection and Conservation Strategy. Agric. Nat. Resour. 41, 7–16.

[B10] Bisht M. S. Mukai Y. (2002). Genome Organization and Polyploid Evolution in the Genus *Eleusine* (Poaceae). Plant Syst. Evol. 233, 243–258. 10.1007/s00606-002-0201-5

[B11] Bisht M. S. Mukai Y. (2001). Genomic *In Situ* Hybridization Identifies Genome Donor of finger Millet (*Eleusine Coracana*). Theor. Appl. Genet. 102, 825–832. 10.1007/s001220000497

[B69] Bolger A. M. Lohse M. Usadel B. (2014). Trimmomatic: A Flexible Trimmer for Illumina Sequence Data. Bioinformatics 30, 2114–2120. 24695404 10.1093/bioinformatics/btu170 PMC4103590

[B12] Brhane H. Haileselassie T. Tesfaye K. Hammenhag C. Ortiz R. Abreha K. B. (2021). Novel Expressed Sequence Tag-Derived and Other Genomic Simple Sequence Repeat Markers Revealed Genetic Diversity in Ethiopian Finger Millet Landrace Populations and Cultivars. Front. Plant Sci. 12. 10.3389/fpls.2021.735610 PMC8495221 34630485

[B13] Chao S. Zhang W. Akhunov E. Sherman J. Ma Y. Luo M.-C. (2009). Analysis of Gene-Derived SNP Marker Polymorphism in US Wheat (*Triticum aestivum* L.) Cultivars. Mol. Breed. 23, 23–33. 10.1007/s11032-008-9210-6

[B14] Chethan S. Malleshi N. G. (2007). Finger Millet Polyphenols: Characterization and Their Nutraceutical Potential. Am. J. Food Tech. 2, 582–592. 10.3923/ajft.2007.582.592

[B15] Cooke T. F. Yee M.-C. Muzzio M. Sockell A. Bell R. Cornejo O. E. (2016). GBStools: a Statistical Method for Estimating Allelic Dropout in Reduced Representation Sequencing Data. PLoS Genet. 12, e1005631. 10.1371/journal.pgen.1005631 26828719 PMC4734769

[B17] Delhaize E. Ryan P. R. (1995). Aluminum Toxicity and Tolerance in Plants. Plant Physiol. 107, 315. 10.1104/pp.107.2.315 12228360 PMC157131

[B18] Dida M. M. Wanyera N. Dunn M. L. H. Bennetzen J. L. Devos K. M. (2008). Population Structure and Diversity in finger Millet (*Eleusine Coracana*) Germplasm. Trop. Plant Biol. 1, 131–141. 10.1007/s12042-008-9012-3

[B19] Eekhout T. Larsen P. De Veylder L. (2017). Modification of DNA Checkpoints to Confer Aluminum Tolerance. Trends Plant Science 22, 102–105. 10.1016/j.tplants.2016.12.003 28065410

[B20] Elshire R. J. Glaubitz J. C. Sun Q. Poland J. A. Kawamoto K. Buckler E. S. (2011). A Robust, Simple Genotyping- By-Sequencing (GBS) Approach for High Diversity Species. PLoS One 6, e19379. 10.1371/journal.pone.0019379 21573248 PMC3087801

[B21] Enyew M. Feyissa T. Carlsson A. S. Tesfaye K. Hammenhag C. Geleta M. (2021). Genetic Diversity and Population Structure of Sorghum [*Sorghum Bicolor* (L.) Moench] Accessions as Revealed by Single Nucleotide Polymorphism Markers. Fronters Plant Sci. 12, 799482. 10.3389/fpls.2021.799482 PMC8766336 35069657

[B22] Erenso D. Asfaw A. Taye T. Tesso T. (2007). “Genetic Resources, Breeding and Production of Millets in Ethiopia,” in Organizing Committee of the International Conference on New Approaches to, Bern, Switzerland, 19-21 September 2007, 43–56. Proceedings of an International Conference.( New Approaches to Plant Breeding of Orphan Crops in Africa

[B23] Excoffier L. Lischer H. E. L. (2010). Arlequin Suite Ver 3.5: a New Series of Programs to Perform Population Genetics Analyses under Linux and Windows. Mol. Ecol. Resour. 10, 564–567. 10.1111/j.1755-0998.2010.02847.x 21565059

[B24] Fakrudin B. Shashidhar H. Kulkarni R. Hittalmani S. (2004). Genetic Diversity Assessment of finger Millet, *Eleusine Coracana* (Gaertn), Germplasm through RAPD Analysis. PGR Newslett 138, 50–54.

[B25] Faostat (2020). Statistical Databases and Data-Sets of the Food and Agriculture Organization of the United Nations.

[B26] Garrison E. Marth G. (2012). Haplotype-based Variant Detection from Short-Read Sequencing. *arXiv preprint arXiv:1207.3907* .

[B27] Geleta M. Gustafsson C. Glaubitz J. Ortiz R. (2020). High-density Genetic Linkage Mapping of Lepidium Based on Genotype-By-Sequencing and Segregating Contig Tag Haplotypes. Front. Plant Sci. 11, 448. 10.3389/fpls.2020.00448 32425961 PMC7204607

[B28] Gimode D. Odeny D. A. De Villiers E. P. Wanyonyi S. Dida M. M. Mneney E. E. (2016). Identification of SNP and SSR Markers in finger Millet Using Next Generation Sequencing Technologies. PLoS one 11, e0159437. 10.1371/journal.pone.0159437 27454301 PMC4959724

[B29] Goron T. L. Raizada M. N. (2015). Genetic Diversity and Genomic Resources Available for the Small Millet Crops to Accelerate a New Green Revolution. Front. Plant Sci. 6. 10.3389/fpls.2015.00157 PMC4371761 25852710

[B30] Hammenhag C. Saripella G. V. Ortiz R. Geleta M. (2020). QTL Mapping for Domestication-Related Characteristics in Field Cress (*Lepidium Campestre*)—a Novel Oil Crop for the Subarctic Region. Genes 11, 1223. 10.3390/genes11101223 PMC7603098 33086591

[B31] Hildebrand C. E. Torney D. V. Wagner R. P. (1994). “Informativeness of Polymorphic DNA Markers,” in, The Human Genome Project: Decipering the Blueprint of Heredity. Editor Cooper N. G. (Mill Valley, CA, USA: University Science Books), 100–102.

[B32] Hilu K. W. De Wet J. (1976). Domestication of *Eleusine Coracana* . Econ. Bot. 30, 199–208. 10.1007/bf02909728

[B33] Hittalmani S. Mahesh H. Shirke M. D. Biradar H. Uday G. Aruna Y. (2017). Genome and Transcriptome Sequence of finger Millet (*Eleusine Coracana* (L.) Gaertn.) Provides Insights into Drought Tolerance and Nutraceutical Properties. BMC Genomics 18, 465. 10.1186/s12864-017-3850-z 28619070 PMC5472924

[B34] Huang Y.-F. Poland J. A. Wight C. P. Jackson E. W. Tinker N. A. (2014). Using Genotyping-By-Sequencing (GBS) for Genomic Discovery in Cultivated Oat. PloS one 9, e102448. 10.1371/journal.pone.0102448 25047601 PMC4105502

[B35] Kebede D. Dagnachew L. Megersa D. Chemeda B. Girma M. Geleta G. (2019). Genotype by Environment Interaction and Grain Yield Stability of Ethiopian Black Seeded finger Millet Genotypes. Afr. Crop Sci. J. 27. 10.4314/acsj.v27i2.12

[B36] Kopelman N. M. Mayzel J. Jakobsson M. Rosenberg N. A. Mayrose I. (2015). Clumpak: a Program for Identifying Clustering Modes and Packaging Population Structure Inferences across K. Mol. Ecol. Resour. 15, 1179–1191. 10.1111/1755-0998.12387 25684545 PMC4534335

[B37] Kopittke P. M. Blamey F. P. C. (2016). Theoretical and Experimental Assessment of Nutrient Solution Composition in Short-Term Studies of Aluminium Rhizotoxicity. Plant and Soil 406, 311–326. 10.1007/s11104-016-2890-5

[B38] Krishna T. P. A. Maharajan T. Antony David R. H. Ramakrishnan M. Ceasar S. A. Duraipandiyan V. (2018). Microsatellite Markers of finger Millet (Eleusine Coracana (L.) Gaertn) and Foxtail Millet (*Setaria Italica* (L.) Beauv) Provide Resources for Cross-Genome Transferability and Genetic Diversity Analyses in Other Millets. Biocatal. Agric. Biotechnol. 16, 493–501. 10.1016/j.bcab.2018.09.009

[B39] Kumar A. Sharma D. Tiwari A. Jaiswal J. Singh N. Sood S. (2016a). Genotyping-by-sequencing Analysis for Determining Population Structure of finger Millet Germplasm of Diverse Origins. The plant genome 9, 1–15. 10.3835/plantgenome2015.07.0058 27898819

[B40] Kumar S. Banks T. W. Cloutier S. (20122012). SNP Discovery through Next-Generation Sequencing and its Applications. Int. J. Plant genomics. 10.1155/2012/831460 PMC3512287 23227038

[B41] Kumar S. Stecher G. Tamura K. (2016b). MEGA7: Molecular Evolutionary Genetics Analysis Version 7.0 for Bigger Datasets. Mol. Biol. Evol. 33, 1870–1874. 10.1093/molbev/msw054 27004904 PMC8210823

[B42] Li H. Durbin R. (2009). Fast and Accurate Short Read Alignment with Burrows–Wheeler Transform. bioinformatics 25, 1754–1760. 10.1093/bioinformatics/btp324 19451168 PMC2705234

[B43] Li Y. L. Liu J. X. (2018). StructureSelector: A Web‐based Software to Select and Visualize the Optimal Number of Clusters Using Multiple Methods. Mol. Ecol. Resour. 18, 176–177. 10.1111/1755-0998.12719 28921901

[B44] Lule D. De Villiers S. Fetene M. Odeny D. A. Rathore A. Das R. R. (2018). Genetic Diversity and Association Mapping of Ethiopian and Exotic finger Millet Accessions. Crop Pasture Sci. 69, 879–891. 10.1071/CP18175

[B45] Molla F. (2010). Genotype X Environment Interaction and Stability Analyses of Yield and Yield Related Traits of finger Millet (Eleusine Coracana (L) Gaertn) Varieties in North Western Ethiopia. Haramaya, Ethiopia: M. Sc. thesis presented to the School of Graduate Studies of Haramaya University.

[B46] Mukarumbwa P. Mushunje A. (2010). Potential of Sorghum and finger Millet to Enhance Household Food Security in Zimbabwe's Semi-arid Regions: A Review. doi. 10.22004/ag.econ.96430

[B47] Nakarani U. M. Singh D. Suthar K. P. Karmakar N. Faldu P. Patil H. E. (2021a). Nutritional and Phytochemical Profiling of Nutracereal finger Millet (*Eleusine Coracana* L.) Genotypes. Food Chem. 341, 128271. 10.1016/j.foodchem.2020.128271 33166822

[B16] National Research Council (1996). Lost Crops of Africa: Volume I: Grains. Washington, D.C.: National Academies Press. 10.17226/2305

[B48] Nei M. (1972). Genetic Distance between Populations. The Am. Naturalist 106, 283–292. 10.1086/282771

[B49] Nirgude M. Babu B. K. Shambhavi Y. Singh U. Upadhyaya H. Kumar A. (2014). Development and Molecular Characterization of Genic Molecular Markers for Grain Protein and Calcium Content in finger Millet (*Eleusine Coracana* (L.) Gaertn.). Mol. Biol. Rep. 41, 1189–1200. 10.1007/s11033-013-2825-7 24477581

[B50] Osorio-Guarín J. A. Berdugo-Cely J. A. Coronado-Silva R. A. Baez E. Jaimes Y. Yockteng R. (2020). Genome-wide Association Study Reveals Novel Candidate Genes Associated with Productivity and Disease Resistance to Moniliophthora Spp. In Cacao (*Theobroma Cacao* L.). G3: Genes, Genomes, Genet. 10, 1713–1725. 10.1534/g3.120.401153 PMC7202020 32169867

[B51] Osterman J. Hammenhag C. Ortiz R. Geleta M. (2021). Insights into the Genetic Diversity of Nordic Red clover (*Trifolium Pratense*) Revealed by SeqSNP-Based Genetic Markers. Front. Plant Sci. 12, 748750. 10.3389/fpls.2021.748750 34759943 PMC8574770

[B52] Pandian S. Satish L. Rameshkumar R. Muthuramalingam P. Rency A. S. Rathinapriya P. (2018). Analysis of Population Structure and Genetic Diversity in an Exotic Germplasm Collection of Eleusine Coracana (L.) Gaertn. Using Genic-SSR Markers. Gene 653, 80–90. 10.1016/j.gene.2018.02.018 29428798

[B53] Park Y.-J. Dixit A. Ma K.-H. Lee J.-K. Lee M.-H. Chung C.-S. (2008). Evaluation of Genetic Diversity and Relationships within an On-Farm Collection of *Perilla Frutescens* (L.) Britt. Using Microsatellite Markers. Genet. Resour. Crop Evol. 55, 523–535. 10.1007/s10722-007-9258-x

[B54] Peakall R. Smouse P. E. (2006). GENALEX 6: Genetic Analysis in Excel. Population Genetic Software for Teaching and Research. Mol. Ecol. Notes 6, 288–295. 10.1111/j.1471-8286.2005.01155.x PMC3463245 22820204

[B55] Poland J. A. Brown P. J. Sorrells M. E. Jannink J.-L. (2012). Development of High-Density Genetic Maps for Barley and Wheat Using a Novel Two-Enzyme Genotyping-By-Sequencing Approach. PloS one 7, e32253. 10.1371/journal.pone.0032253 22389690 PMC3289635

[B56] Poland J. A. Rife T. W. (2012). Genotyping‐by‐sequencing for Plant Breeding and Genetics. The Plant Genome 5. 10.3835/plantgenome2012.05.0005

[B57] Pritchard J. K. Stephens M. Donnelly P. (2000). Inference of Population Structure Using Multilocus Genotype Data. Genetics 155, 945–959. 10.1093/genetics/155.2.945 10835412 PMC1461096

[B58] Puechmaille S. J. (2016). The Program Structure Does Not Reliably Recover the Correct Population Structure when Sampling Is Uneven: Subsampling and New Estimators Alleviate the Problem. Mol. Ecol. Resour. 16, 608–627. 10.1111/1755-0998.12512 26856252

[B59] Qi P. Gimode D. Saha D. Schröder S. Chakraborty D. Wang X. (2018). UGbS-Flex, a Novel Bioinformatics Pipeline for Imputation-free SNP Discovery in Polyploids without a Reference Genome: finger Millet as a Case Study. BMC Plant Biol. 18, 1–19. 10.1186/s12870-018-1316-3 29902967 PMC6003085

[B60] Ramakrishnan M. Ceasar S. A. Duraipandiyan V. Al-Dhabi N. A. Ignacimuthu S. (2016). Assessment of Genetic Diversity, Population Structure and Relationships in Indian and Non-Indian Genotypes of finger Millet (*Eleusine Coracana* (L.) Gaertn) Using Genomic SSR Markers. Springerplus 5. 10.1186/s40064-015-1626-y PMC4749518 26900542

[B61] Rozas J. Sánchez-Delbarrio J. C. Messeguer X. Rozas R. (2003). DnaSP, DNA Polymorphism Analyses by the Coalescent and Other Methods. Bioinformatics 19, 2496–2497. 10.1093/bioinformatics/btg359 14668244

[B62] Shete S. Tiwari H. Elston R. C. (2000). On Estimating the Heterozygosity and Polymorphism Information Content Value. Theoretical Population Biology 57, 265–271. 10.1006/tpbi.2000.1452 10828218

[B63] Sri S. Dida M. M. Gale M. D. Devos K. M. (2007). Comparative Analyses Reveal High Levels of Conserved Colinearity between the finger Millet and rice Genomes. Theor. Appl. Genet. 115, 489–499. 10.1007/s00122-007-0582-5 17619853

[B64] Tiwari A. Sharma D. Sood S. Jaiswal J. P. Pachauri S. P. Ramteke P. W. (2020). Genome-wide Association Mapping for Seed Protein Content in finger Millet (*Eleusine Coracana*) Global Collection through Genotyping by Sequencing. J. Cereal Sci. 91, 102888. 10.1016/j.jcs.2019.102888

[B65] Tsehay S. Ortiz R. Johansson E. Bekele E. Tesfaye K. Hammenhag C. (2020). New Transcriptome-Based SNP Markers for Noug (*Guizotia Abyssinica*) and Their Conversion to KASP Markers for Population Genetics Analyses. Genes 11, 1373. 10.3390/genes11111373 PMC7709008 33233626

[B66] Tsehaye T. Kebebew F. (2002). Morphological Diversity and Geographic Distribution of Adaptive Traits in finger Millet (*Eleusine Coracana* (L.) Gaertn.[Poaceae]) Populations from Ethiopia. Ethiopian J. Biol. Sci. 1, 37–62.

[B67] Upadhyaya H. Gowda C. Reddy V. G. (2007). Morphological Diversity in finger Millet Germplasm Introduced from Southern and Eastern Africa. J. SAT Agric. Res. 3, 1–3.

[B68] Zhang H. Jiang Z. Qin R. Zhang H. Zou J. Jiang W. (2014). Accumulation and Cellular Toxicity of Aluminum in Seedling of Pinus Massoniana. BMC Plant Biol. 14, 1–16. 10.1186/s12870-014-0264-9 25267390 PMC4189629

