# Supplementary figures and images for: Novel GBS-Based SNP Markers for Finger Millet and Their Use in Genetic Diversity Analyses
Source: Front Genet. 2022 Apr 26;13:848627. doi: 10.3389/fgene.2022.848627 (PMC9090224; doi:10.3389/fgene.2022.848627)

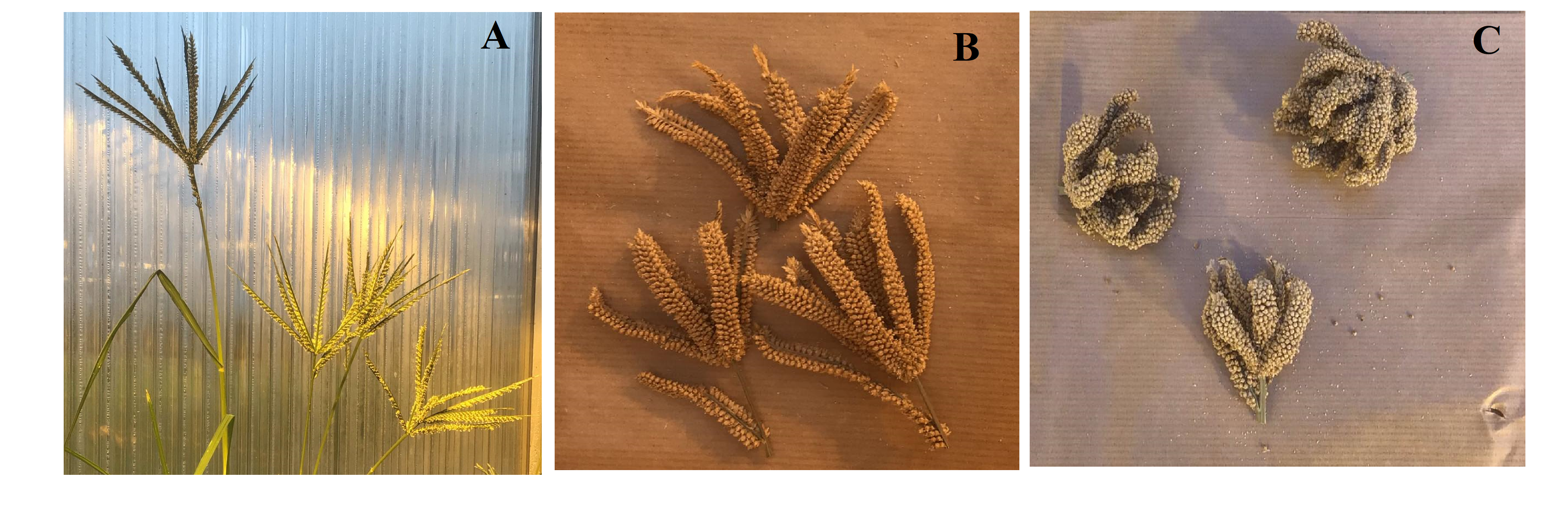

Supplement: Supplementary file 1 [file Image3.TIFF]

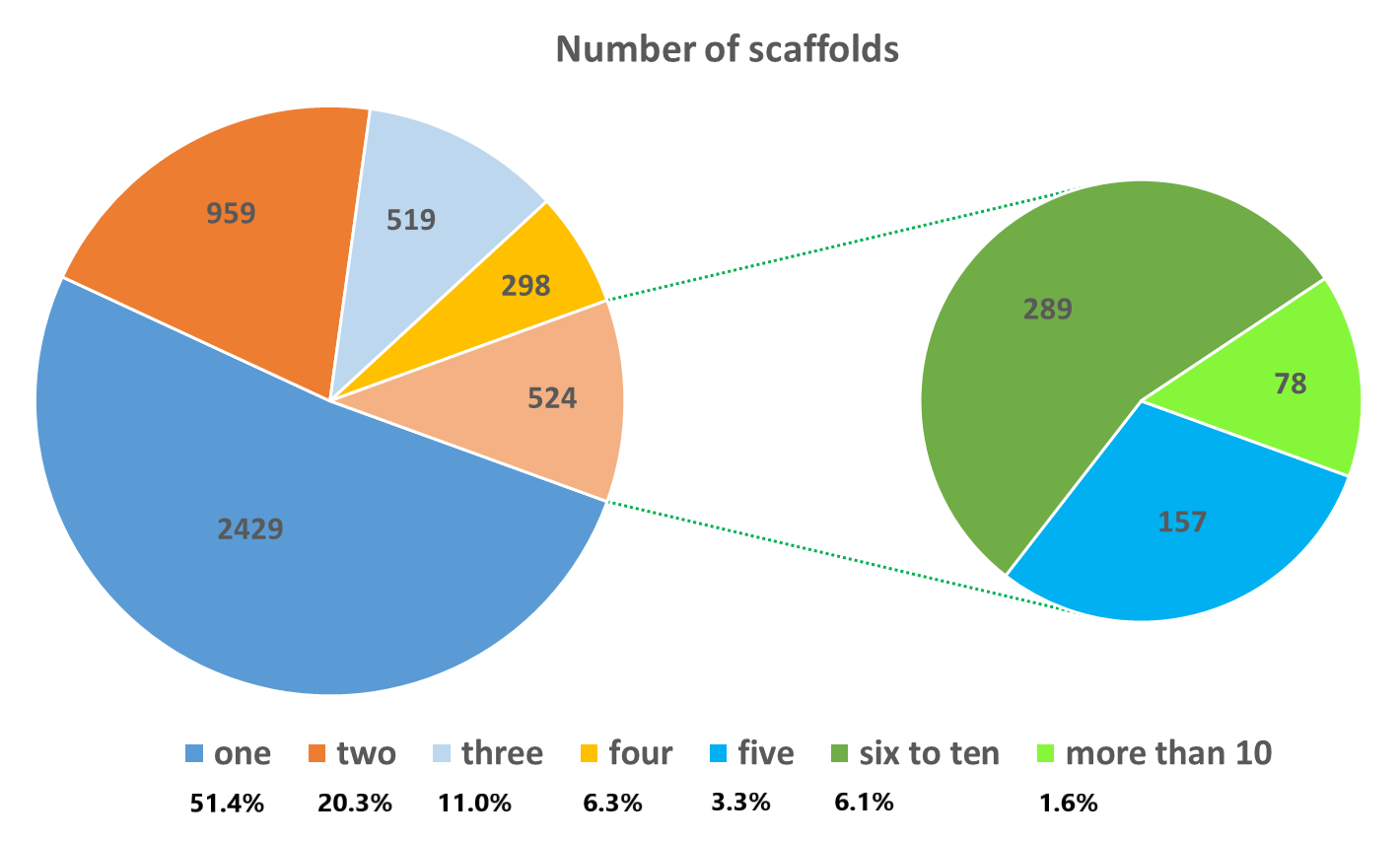

Supplement: Supplementary file 3 [file Image1.TIFF]

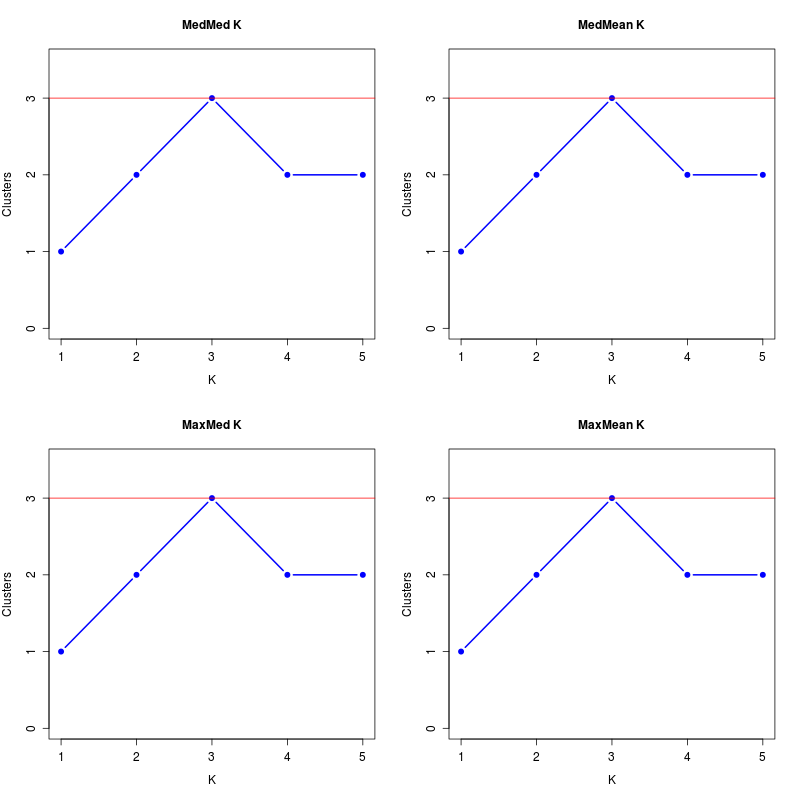

Supplement: Supplementary file 4 [file Image5.TIFF]

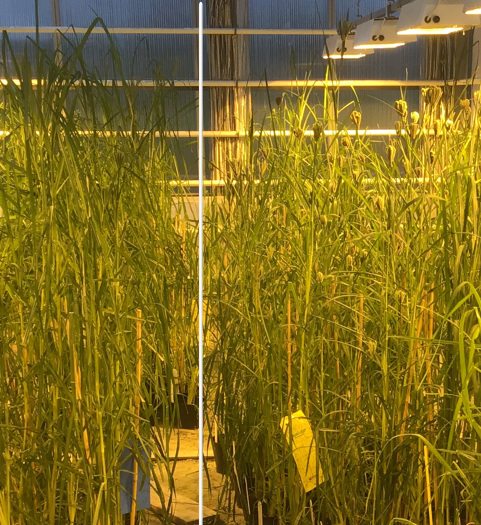

Supplement: Supplementary file 6 [file Image2.TIFF]

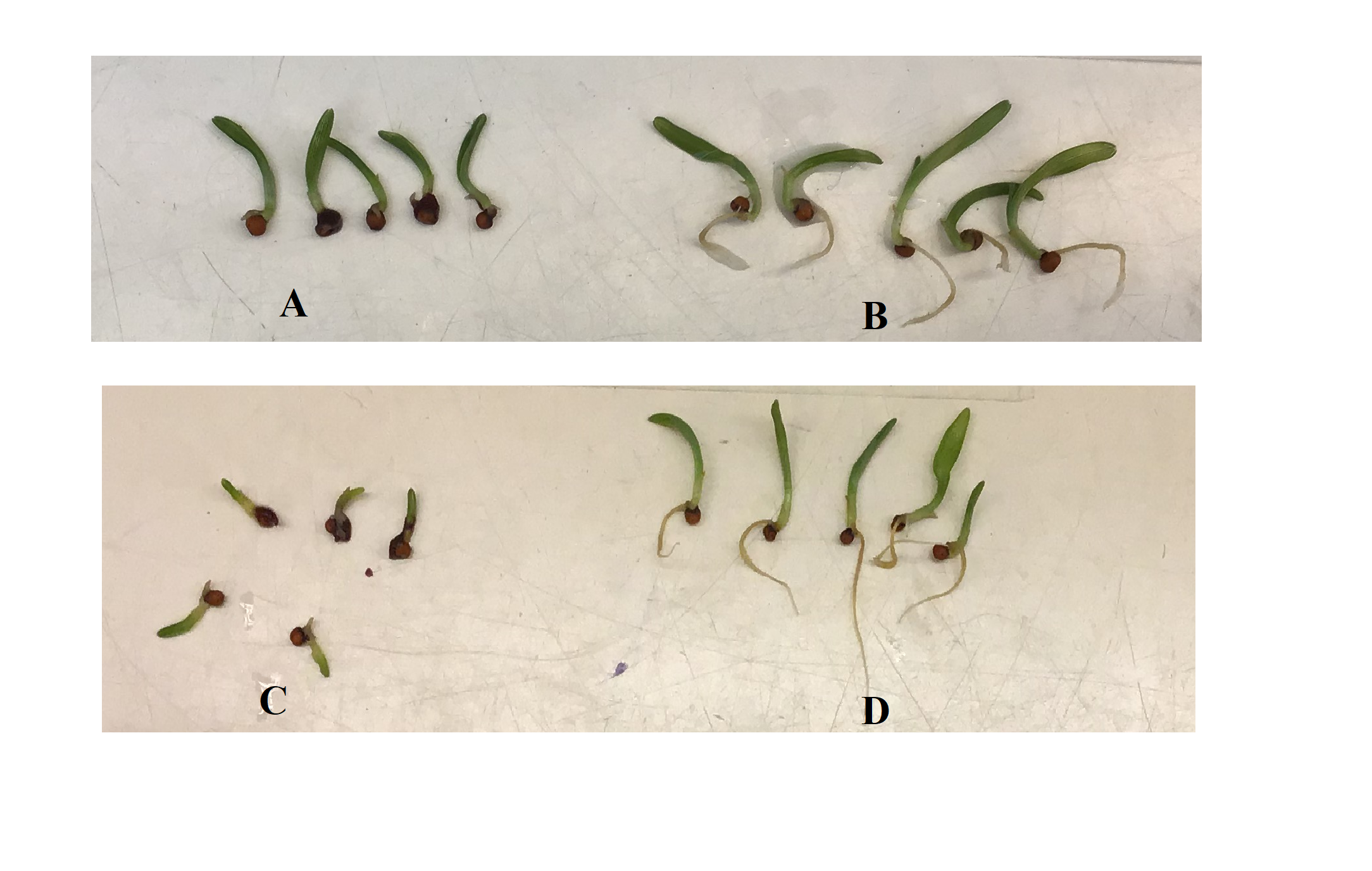

Supplement: Supplementary file 7 [file Image4.TIFF]
